# Methylglyoxal Attenuates *Mycobacterium avium subspecies paratuberculosis* (MAP)-Induced Pro-Inflammatory Macrophage Programming Associated with NRF-2 Antioxidant Responses and Reduced MCT4/Lactate-Linked Inflammatory Markers

**DOI:** 10.3390/ijms27156940

**Published:** 2026-08-02

**Authors:** Heba R. Alrefaey, Saleh A. Naser

**Affiliations:** Burnett School of Biomedical Sciences, College of Medicine, University of Central Florida, Orlando, FL 32827, USA; heba.alrefaey@ucf.edu

**Keywords:** Crohn’s disease, methylglyoxal, *Mycobacterium avium paratuberculosis* (MAP), cytokines, monocarboxylate transporters (MCT-4), nuclear factor erythroid 2-related factor 2 (Nrf-2)

## Abstract

Crohn’s disease (CD) is a chronic inflammatory bowel disease with a rising incidence and prevalence worldwide. It is associated with *Mycobacterium avium subspecies paratuberculosis* (MAP). Current CD treatment strategies are based on anti-inflammatory therapies, including anti-TNF-α drugs. These treatment options provide short-term benefits and are associated with numerous side effects in CD patients. Manuka honey is distinguished from other honey by its high content of methylglyoxal (MGO). MGO, a reactive metabolite, is also generated endogenously in macrophages during infection through glycolysis; however, the amount is insufficient to neutralize the ongoing infection and subsequent tissue damage. This study examined whether exogenous, low-dose MGO can modulate MAP-driven inflammatory and glycolysis- and lactate-associated markers in infected macrophages. THP-1 macrophages were infected with the CD-associated MAP strain and then treated with MGO doses at defined time intervals. We measured markers of M1-/M2-like phenotype polarization, monocarboxylate transporters, lactate export, antioxidant responses, cytokines, and selected glycolysis- and lactate-associated markers at both the mRNA and protein levels. MGO reduced M1 signaling markers *CXCL10* (*p* < 0.05), *TNF-α* (*p* < 0.0001), *IL-1β* (*p* < 0.01), and *IL-6* (*p* < 0.0001). Simultaneously, MGO promoted M2 shift, elevating *CD206* by 1.20-fold and *IL-10* by 7-fold. Low-dose MGO administration was associated with increases in *Nrf-2* (1.4-fold), *HO-1* (1.4-fold), and *IL-1Ra* (1.5-fold), while the pro-inflammatory cytokines decreased. Metabolically, MGO downregulated *MCT4* (*p* < 0.01) and reduced lactate export by 30%. These changes were coupled with higher *PHD2* (1.4-fold) and decreases in *GLUT1* (0.9-fold), *PKD1* (0.8-fold), and *IL-1β*, consistent with attenuated glycolysis- and lactate-associated inflammatory signaling. These results suggest that hormetic concentration of MGO mitigates MAP-induced inflammatory activation while altering glycolysis- and lactate-related signaling markers in infected macrophages. Most importantly, we unraveled the predicted molecular mechanism by which MGO suppresses inflammation and modulates oxidative damage.

## 1. Introduction

Inflammatory Bowel Disease (IBD) is a chronic gastrointestinal inflammatory illness that affects both men and women at a young age, and it includes Crohn’s disease (CD) or ulcerative colitis (UC) [1,2]. Both are characterized by chronic bowel relapse, where CD can strike any part of the gastrointestinal tract non-continuously, involving the terminal ileum, cecum, and colon [3]. According to the US Census, a total of 1.1 million patients are suffering from CD [4]. Although the exact etiology is unknown, it is likely an interplay of genetic susceptibility, immune dysregulation, and environmental factors [5]. Researchers have isolated *Mycobacterium avium paratuberculosis* (MAP) from the blood, intestinal tissues, and body fluids of individuals with CD [6,7,8]. Several systematic and meta-analyses have evaluated the association between MAP and CD using culture-based, polymerase chain reaction (PCR)-based, and other nucleic acid detection approaches. These analyses support a positive association between MAP detection and CD [9,10]. Most recent research further highlights MAP as a biologically relevant microbial factor in a subset of CD patients and supports the development of therapeutic strategies that account for MAP-associated host–pathogen interactions [11,12]. Interestingly, MAP blocks phagolysosomal degradation and shifts macrophages toward M1, releasing pro-inflammatory cytokines, such as *IL-6* and *TNF-α*, which exacerbate inflammation and damage to the intestinal epithelial barrier [13,14]. Anti-*TNF* therapy represents an important treatment strategy even though a proportion of patients exhibit either a limited response or adverse outcomes. Anti-TNF-α therapies are reported to provide short-term effectiveness, and many patients suffer from intolerance within the first year. In such cases, patients may need to switch to a different therapeutic class, such as anti-integrins, anti-interleukins, and JAK inhibitors [15,16,17]. In vitro studies have further demonstrated that blocking the *IL-6* receptor can exacerbate epithelial injury induced by MAP [14]. Corticosteroids, such as budesonide, are commonly used to treat active intestinal diseases. However, due to the complexity of targeting inflammatory pathways in CD, corticosteroids are not consistently effective in promoting mucosal healing [5]. Ultimately, patients with CD often require long-term medications and, in some cases, demand hospitalization and surgery, which will consequently impose substantial health care burdens. These findings highlight the need for alternative strategies that reduce pathological inflammation without compromising host defense. Metabolic reprogramming is central to immune-cell activation and differentiation, not only because it supplies energy and biosynthetic intermediates, but also because metabolic products can act as signaling mediators that shape immune responses [18]. In this context, selected endogenous metabolites, such as methylglyoxal (MGO), may offer a promising strategy for modulating MAP-associated inflammatory responses associated with CD by targeting the metabolic signals that shape macrophage activation.

Some studies have shown that during bacterial infection, activated macrophages shift from anaerobic to aerobic glycolysis, a metabolic pathway in which cells accelerate ATP production by relying on glycolysis rather than mitochondrial oxidative phosphorylation [19,20,21]. An activated macrophage extensively relies on increased glycolytic flux, which is stabilized by the transcription of *HIF-1α* (hypoxia-inducible factor-1 alpha) [22]. HIF-1α boosts the transcription of metabolic genes that sustain glycolytic flux, including the inducible hexokinase isoform HK2 [23,24]. Under hypoxic conditions, glycolytic enzymes such as lactate dehydrogenase (LDH), phosphofructokinase (PFK), and hexokinase II (HKII) are upregulated [25]. As shown previously, macrophage HIF-1α drives pyruvate dehydrogenase kinase 1 (PDK-1) expression, which restrains glucose oxidation by inhibiting pyruvate dehydrogenase (PDH) and thereby limiting pyruvate entry into the tricarboxylic acid cycle (TCA) [26]. Consequently, the overexpressed LDH catalyzes the conversion of pyruvate into lactate, which is then exported extracellularly through monocarboxylate transporters (MCTs), thereby contributing to the extracellular acidity [27]. Tan et al. demonstrated that MCT4 is upregulated in macrophages in response to activation of TLR2 or TLR4 agonists, driven by elevated glycolytic demand, thereby increasing lactate production [28]. Interestingly, research by Feng et al. showed that lactate can directly bind to and inhibit the activity of prolyl hydroxylase domain-containing protein 2 (PHD2), the enzyme that hydroxylates HIF-1α. Therefore, lactate elicits the stabilization of HIF-1α, which in turn transactivates IL-1β in response to inflammatory stimuli [29]. Collectively, HIF-1α and MCT-4 promote the transcription of inflammatory cytokine genes, most prominently interleukin (IL)-1β [22,30].

Methylglyoxal (MGO) is a reactive metabolite formed during glycolysis or as a byproduct of lipid and protein metabolism [31,32]. MGO is a vital and integral constituent of Manuka honey, a type of honey derived from Leptospermum scoparium trees, which is well-known for its antimicrobial and numerous therapeutic and medicinal beneficial properties [33,34,35,36]. Anaya-Sanchez et al. showed that infected macrophages produce MGO and may play a role as an innate antibacterial effector [37]. Another study reported that mice given MGO orally over a long period of time exhibited a mild increase in plasma MGO, lived longer, had fewer age-related solid tumors, and did not develop diabetes or kidney dysfunction [38]. MGO has also been studied in cancer models, where its biological activity appears to be dose-dependent and linked to altered glycolytic metabolism [39]. In addition, the study supports the broader relevance of MGO as an immunometabolic molecule, while also emphasizing the importance of dose selection because excessive MGO accumulation can contribute to carbonyl stress in inflammatory conditions such as sepsis [40]. MGO can react with proteins, nucleotides, and basic phospholipids by glycation, leading to the formation of the harmful advanced glycation end products (AGEs) [41]. The most abundant quantitative and functional AGEs in physiological systems are arginine-derived hydroimidazolones (MG-H1) [42]. Hormesis is a process in which exposure of cells to low doses of chemical agents or metabolites that are detrimental at higher doses induces an adaptive, cytoprotective response [43]. This study is the first to evaluate the hormetic effect of MGO on macrophages during MAP infection and to focus on the molecular signaling mechanisms underlying MGO’s ability to reverse inflammation in an in vitro CD-like system.

## 2. Results

### 2.1. MGO Has an Innate Antibacterial Effector in MAP-Infected THP-1 Macrophages

**Figure 1:**
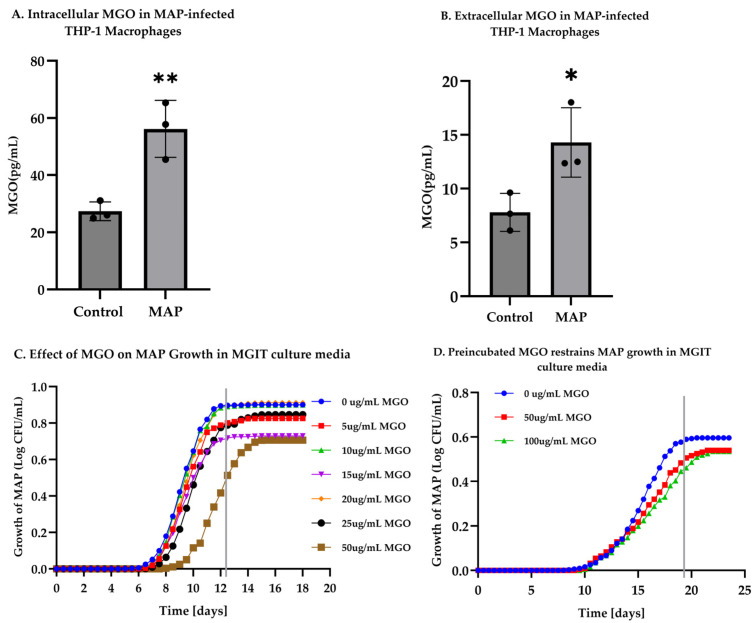
MGO has an innate antibacterial effector in MAP-infected THP-1 macrophages. M1-polarized THP-1 macrophages produce elevated levels of MGO intracellularly (**A**) and extracellularly (supernatant, i.e., serum-free media) (**B**). The data are from three independent experiments. Standard deviation is shown for all experiments, and *p* values were calculated using an unpaired, two-tailed *t*-test; *n* = 3; * *p* < 0.05, ** *p* < 0.01 are considered significant. The antimicrobial effect of MGO on MAP. (**C**) MGIT tubes were initially inoculated with MAP, treated with different concentrations of MGO (0, 5, 10, 15, 20, 25, and 50 μg/mL), and monitored daily for growth. (**D**) The chemical stability and ability to sustain antibacterial activity of MGO were evaluated by pretreating MGIT tubes with MGO at concentrations of 0, 50, and 100 μg/mL. Then, the tubes were incubated under standard conditions for 30 days before being inoculated with MAP.

Intracellular MGO levels were significantly elevated in MAP-infected THP-1 macrophages, with 2.05 ± 0.21-fold compared to the uninfected control (Figure 1A; *p* < 0.01, *n* = 3), and the extracellular levels of MGO increased by 1.83 ± 0.24 (Figure 1B; *p* < 0.05, *n* = 3). We investigated the antibacterial effect of MGO on MAP in MGIT media, and MAP growth units (Log CFU/mL) were measured daily for 18 days. Our findings demonstrated that increasing MGO concentrations produced a dose-dependent inhibition in MAP growth, reflected by a delayed lag-to-exponential transition and a reduced maximal growth plateau. A total of 50 µg/mL MGO shows the highest percentage of CFU growth inhibition on day 12 (98.8%) (Figure 1C). Next, we tested whether MGO remains active and maintains its protective antibacterial activity by pretreating MGIT tubes with MGO at 0, 50, and 100 μg/mL. Then, the tubes were incubated under standard conditions for 30 days before being inoculated with MAP. As shown in Figure 1D, MAP growth was still curtailed despite the month-long preincubation. At day 19 post-infection, inhibition reached 81.2% with 50 µg/mL MGO and 90.6% with 100 µg/mL MGO relative to untreated controls.

### 2.2. Cytotoxicity and Viability Assessment of MGO in MAP-Infected THP-1 Macrophages

**Figure 2:**
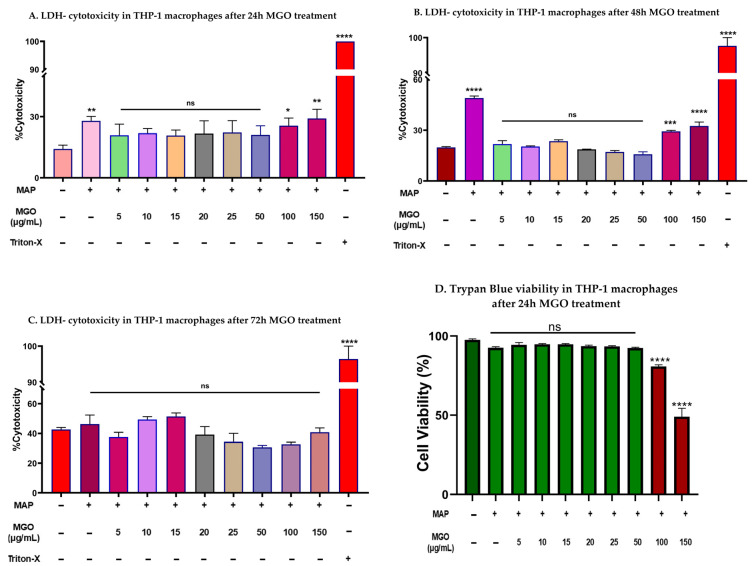
Cytotoxicity and viability assessment of MAP-infected THP-1 macrophages following MGO treatment. THP-1 macrophages were infected with MAP and treated with increasing concentrations of MGO. (**A**–**C**) LDH-Glo cytotoxicity assay after 24 h, 48 h, and 72 h of MGO treatment, respectively. LDH-Glo measures LDH released from membrane-damaged cells and is presented as % cytotoxicity. Triton-X was used as the maximum-lysis positive control. (**D**) Trypan Blue exclusion assay showing cell viability (%) after 24 h of MGO treatment, the primary time point used for downstream mechanistic experiments. Data are presented as mean ± SEM from three biological replicates (*n* = 3). Statistical significance was assessed using one-way ANOVA followed by multiple comparisons analysis. * *p* < 0.05, ** *p* < 0.01, *** *p* < 0.001, and **** *p* < 0.0001 are considered significant; ns, not statistically significant.

To determine whether MGO treatment caused cell membrane damage in MAP-infected THP-1 macrophages, LDH released into the culture medium was measured using the LDH-Glo cytotoxicity assay. The Trypan Blue exclusion assay was used as a complementary direct viability assay to determine whether the selected MGO concentrations preserved the percentage of viable THP-1 macrophages after 24 h of treatment. The assessment of MGO cytotoxicity illustrated that 5–50 µg/mL MGO did not increase LDH release in THP-1 macrophages infected with MAP at 24 h or 48 h (Figure 2A,B). At 48 h, the control and MAP groups showed a modest increase in cytotoxicity compared with their corresponding 24 h values, with 1.40 ± 0.04-fold and 1.76 ± 0.04-fold increases, respectively (Figure 2A vs. Figure 2B). At 72 h, LDH release increased more broadly across the tested groups, most notably in the untreated control group, which showed 3.00 ± 0.18-fold and 2.14 ± 0.07-fold increases relative to the control groups at 24 h and 48 h, respectively (Figure 2C). Therefore, the apparent decline in the relative cytotoxic effect of 100 and 150 µg/mL MGO at 72 h may be influenced by the elevated baseline LDH release observed in the untreated control group during prolonged culture, rather than indicating complete recovery from high-dose MGO-induced stress. One possible explanation is that prolonged MGO exposure may allow surviving macrophages to activate adaptive detoxification mechanisms, thereby reducing the effective MGO burden over time. These findings suggest that prolonged culture duration contributed to time-dependent membrane stress at later time points and support the selection of 24 h treatment with 5–50 µg/mL MGO as a suitable condition for downstream experiments.

To directly assess cell viability at the main experimental time point, Trypan Blue exclusion was performed after 24 h of MGO treatment. Cell viability remained high, approximately 93–96%, after treatment with 5–50 µg/mL MGO, whereas a marked reduction in viable cells was observed at 100 and 150 µg/mL MGO (Figure 2D). Together, these data indicate that 5–50 µg/mL MGO did not significantly compromise THP-1 macrophage viability under the 24 h treatment condition, supporting the use of 50 µg/mL MGO as the selected working concentration for downstream experiments.

### 2.3. MGO Reduces TNF-α in MAP-Infected THP-1 Macrophages

**Figure 3:**
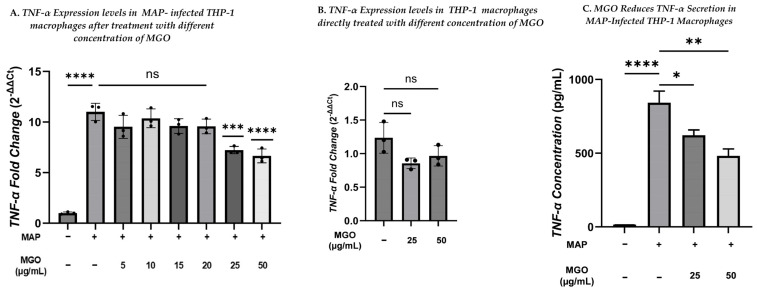
(**A**) Effect of MGO on *TNF-α* in MAP-infected THP-1 macrophages. THP-1 cells were differentiated with PMA for 48 h, infected with MAP strain UCF4 at 1 × 10^7^ CFU/mL for 24 h, and then treated with the indicated MGO concentrations (5–50 µg/mL MGO) for an additional 24 h. *TNF-α* transcripts were quantified by qRT-PCR (*n* = 3). (**B**) *TNF-α* expression in uninfected THP-1 macrophages directly treated with MGO. THP-1 macrophages were treated with 25 or 50 µg/mL MGO for 24 h, followed by RNA extraction and qRT-PCR analysis. TNF-α expression was calculated using the 2^−ΔΔCt^ method. (**C**) MGO reduces TNF-α secretion in MAP-infected THP-1-derived macrophages. THP-1 monocytes were differentiated with PMA, infected with MAP strain UCF4 for 24 h, and subsequently treated with 25 or 50 µg/mL MGO for an additional 24 h. TNF-α concentrations in cell supernatants were measured by ELISA. Data are expressed as mean ± SEM of triplicate samples. Significance was calculated by one-way ANOVA with Bonferroni correction. Data are represented as mean ± SD; *n* = 3. * *p* < 0.05, ** *p* < 0.01, *** *p* < 0.001, and **** *p* < 0.0001 are considered significant. ns: not statistically significant.

*TNF-α* expression in MAP-infected THP-1 macrophages following MGO treatment was measured. As expected, *TNF-α* expression in the MAP-treated group was upregulated (10.96 ± 0.49-fold increase). Treatment with 5–20 µg/mL MGO did not significantly alter *TNF-α* expression levels. In contrast, at 25 and 50 µg/mL MGO, *TNF-α* expression levels were remarkably reduced relative to the untreated MAP-infected group (0.66 ± 0.02-fold; *p* < 0.001, *n* = 3 and 0.61 ± 0.04-fold-decrease; *p* < 0.0001, *n* = 3, respectively (Figure 3A). Therefore, 25 and 50 µg/mL MGO will be used in subsequent experiments.

To determine whether MGO alone induces inflammatory activation, uninfected THP-1 macrophages were treated directly with 25 or 50 µg/mL MGO. Neither concentration significantly increased *TNF-α* expression, indicating that the observed effect of MGO occurred predominantly during MAP-induced inflammatory activation rather than through nonspecific modulation of TNF-α in uninfected macrophages (Figure 3B).

To determine whether the designated concentrations of MGO-mediated reduction in *TNF-α* mRNA expression were also reflected at the protein level, TNF-α secretion was measured by ELISA in cell culture supernatants. MAP infection markedly increased TNF-α secretion to 843.26 ± 78.20 pg/mL, compared with 3.49 ± 0.45 pg/mL in the uninfected control group (*p* < 0.0001). Treatment of MAP-infected macrophages with 25 µg/mL MGO significantly reduced TNF-α secretion to 622.12 ± 35.85 pg/mL (*p* < 0.05 versus MAP), while 50 µg/mL MGO further decreased TNF-α secretion to 481.81 ± 48.16 pg/mL (*p* < 0.01 versus MAP; Figure 3C). Collectively, these findings demonstrate that MGO attenuates MAP-induced TNF-α expression and secretion, supporting its anti-inflammatory activity in MAP-infected THP-1-derived macrophages. Data are presented as mean ± SEM from three biological replicates.

### 2.4. MGO Shifts MAP-Infected THP-1 Macrophages Toward M2 Polarization Through Downregulation of CXCL10

**Figure 4:**
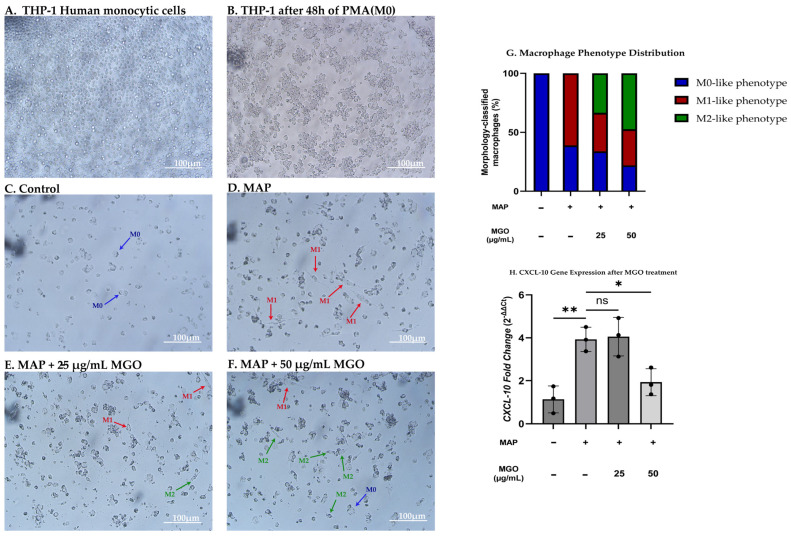
Effect of MAP and MGO on THP-1 macrophages’ M1/M2-like phenotype polarization. THP-1 cells (**A**) before and (**B**) after 48 h of PMA treatment show morphological changes from round to elongated and flattened shapes.THP-1 monocytes were differentiated into macrophages using PMA for 48 h, followed by media replacement. Images were obtained using a light microscope at 10× magnification. Cells were then divided into four groups: (**C**) PMA-differentiated THP-1 cells in fresh media (control). (**D**) Cells infected with MAP for 24 h. (**E**) MAP-infected THP-1 cells treated with 25 µg/mL MGO for 24 h (MAP + 25 µg/mL MGO). (**F**) MAP-infected cells treated with 50 µg/mL MGO for 24 h (MAP + 50 µg/mL MGO). (**G**) Morphological quantification of M0-, M1-, and M2-like macrophage phenotypes following MAP infection and MGO treatment. THP-1-derived macrophages were classified according to their morphological characteristics as M0-like, M1-like, or M2-like in the control, MAP, MAP + 25 µg/mL MGO, and MAP + 50 µg/mL MGO groups. Stacked columns represent the percentage distribution of each phenotype within each experimental condition. (**H**) THP-1 cells were differentiated with PMA for 48 h, infected with MAP for 24 h, and then treated with the indicated MGO concentrations for an additional 24 h. CXCL-10 expression levels were quantified by qRT-PCR (*n* = 3). Data are expressed as mean ± SD of triplicate samples. * *p* < 0.05, ** *p* < 0.01 are considered significant. ns: not statistically significant.

THP-1 macrophages were infected with the MAP for 24 h, treated with 25 and 50 µg/mL MGO for 24 h, and then imaged using bright-field microscopy. Quantitatively, the expression level of *CXCL10* (C-X-C motif chemokine ligand-10) as a marker for M1 macrophage polarization was investigated after treating MAP-infected THP-1 macrophages with 25 and 50 µg/mL MGO for 24 h. The morphologic examination showed that, after PMA differentiation, THP-1 cells shifted from non-adherent monocytes to adherent macrophages, changing from round cells to irregular/semi-rounded forms with greater cell size (Figure 4A,B). In the uninfected group, macrophages exhibited the typical M0 phenotype, characterized by small, semi-rounded, non-polarized cells (Figure 4C). MAP infection induced morphological alterations, including elongated, spindle-like cells and increased cytoplasmic volume, consistent with M1-associated polarization morphology (Figure 4D). Treating MAP-infected THP-1 macrophages with 25 µg/mL MGO resulted in a heterogeneous cell population, with a subset retaining M1-associated features, while the rest of the macrophages displayed a more rounded and spread morphology, suggesting a partial shift toward the M2-associated anti-inflammatory phenotype (Figure 4E). Treatment with 50 µg/mL MGO led to a more uniform morphological response, with reduced branching. Moreover, most cells predominantly adopt a rounded M2-associated morphological anti-inflammatory shift, indicative of cellular remodeling (Figure 4F). Overall, 50 µg/mL MGO appears to favor a more complete transition toward an M2-like phenotype, whereas 25 µg/mL elicits a mixed M1/M2-associated phenotypic response (Figure 4G). To support this, we measured *CXCL-10* in MAP-infected THP-1 macrophages treated with MGO at 25 µg/mL or 50 µg/mL for 24 h. MGO reduced *CXCL10* by 0.492 ± 0.09-fold compared with the untreated group (Figure 4H; *p* < 0.05; *n* = 3), whereas 25 µg/mL had no significant effect. Collectively, although both 25 and 50 µg/mL MGO significantly reduced MAP-induced *TNF-α* mRNA expression, ELISA analysis demonstrated a graded reduction in TNF-α secretion, with 50 µg/mL MGO producing the greater effect. In contrast, significant *CXCL10* downregulation was observed only at 50 µg/mL MGO. These findings indicate marker-specific dose sensitivity and suggest that a higher MGO concentration is required for broader attenuation of MAP-induced M1-associated inflammatory programming.

### 2.5. Effect of MGO on CD206 in MAP-Infected THP-1 Macrophages

**Figure 5:**
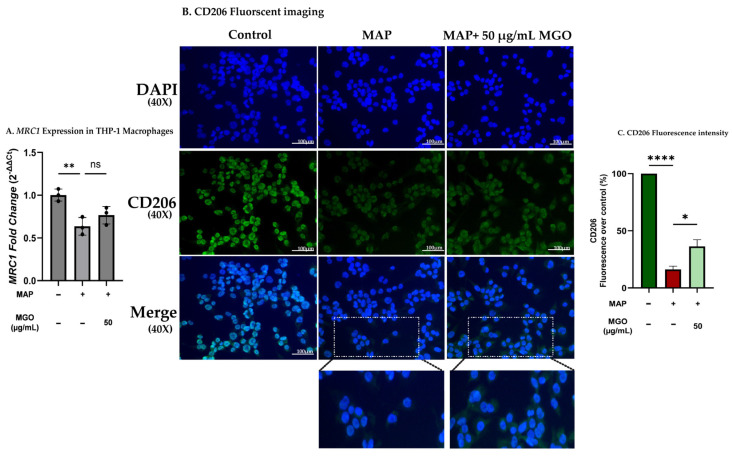
Effect of MGO on CD206 (*MRC1*) expression in THP-1 macrophages infected with MAP. (**A**) THP-1 cells were differentiated into macrophages with PMA, then infected with MAP for 24 h. Following 24 h incubation with 50 µg/mL MGO, RNA was extracted, and *MCR1* mRNA expression was assessed by qRT-PCR. (**B**) Immunofluorescent stain for CD206 expression in THP-1 macrophages infected with MAP, then treated with 50 µg/mL MGO for 24 h. (**C**) Bar graph represents the percentage of CD206 fluorescence intensity quantified from fluorescence microscopy images using ImageJ 1.52a software. Corrected total cell fluorescence (CTCF) was calculated for each image after background correction and expressed as a percentage of fluorescence over the untreated control. Significance was assessed using one-way ANOVA with a post hoc Bonferroni correction. Data are represented as mean ± SEM; *n* = 3. * *p* < 0.05, ** *p* < 0.01, and **** *p* < 0.0001. ns: not statistically significant.

To confirm that MGO induces the shift in infected macrophages toward M2-like polarization, Mannose receptor C-type 1 (*MRC1*), also known as CD206, was evaluated. MGO slightly increased *MRC1* by 1.20 ± 0.09-fold (Figure 5A). Consistently, immunofluorescence staining showed higher CD206 signal in MAP-infected THP-1 cells treated with 50 µg/mL MGO compared with untreated MAP (Figure 5B). Interestingly, these data indicate that MGO promotes an M2-associated phenotype in MAP-infected THP-1 macrophages.

### 2.6. Exogenous MGO Displays Anti-Inflammatory Properties and Diminishes Pro-Inflammatory Cytokines in MAP-Infected THP-1 Macrophages

To assess the role of MGO on pro-inflammatory and anti-inflammatory cytokines, we evaluated the expression levels of NF-κB target genes, including *TNF-α*, *IL-1β*, *IL-6*, and *IL-10*. We tested the impact of MGO on the rapidly induced pro-inflammatory cytokine, *TNF-α*, after 24 h of MGO treatment. While the delayed-expressed cytokines, including *IL-1β* and *IL-6*, were examined after treating infected THP-1 macrophages with MGO for 24 h, 48 h, and 72 h. Additionally, the anti-inflammatory cytokine *IL-10* was also evaluated after 24 h of MGO treatment.

**Figure 6:**
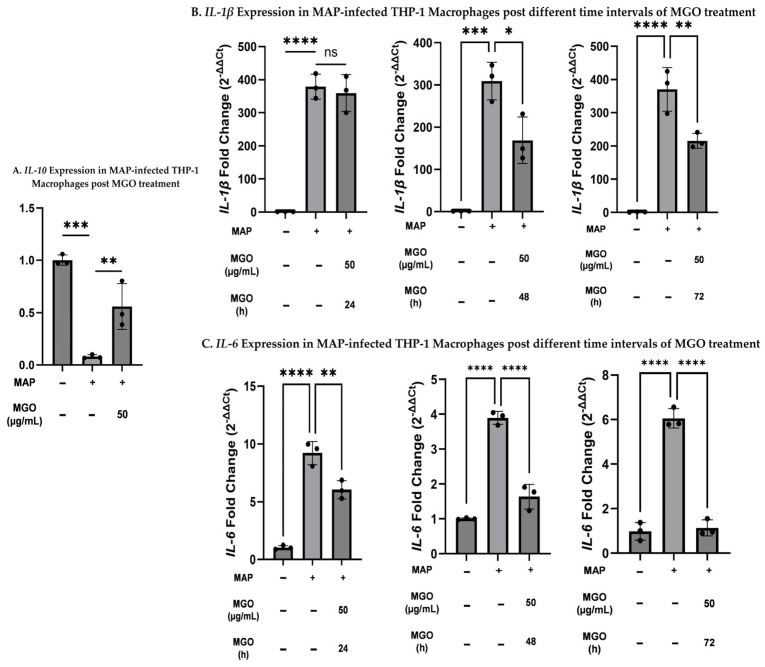
MGO displays anti-inflammatory characteristics in MAP-infected THP-1 macrophages. THP-1 cells were differentiated with PMA, infected with MAP, and then treated with 50 µg/mL MGO. (**A**) Relative *IL-10* Expression. (**B**) THP-1 cells were differentiated with PMA, infected with MAP, and then treated with 50 µg/mL MGO for 24, 48, and 72 h. Then, RNA was collected at each time point and analyzed by qRT-PCR for *IL-1β* expression level. (**C**) qRT-PCR analysis of *IL-6* Expression. Gene expression was calculated using the 2^−ΔΔCt^ method for the MAP-infected and MGO-treated groups. Data represent mean  ±  SEM; significance was calculated by one-way ANOVA with Dunnett’s post hoc test comparing MGO dose to the MAP group. Data shown for at least *n* = 3. * *p* < 0.05, ** *p* < 0.01, *** *p* < 0.001, and **** *p* < 0.0001 are considered significant. ns: not statistically significant.

Our data showed that MGO significantly elevated *IL-10* expression by 6.91 ± 1.56-fold compared with the untreated group (Figure 6A; *p* < 0.01, *n* = 3). MGO had no significant effect on *IL-1β* at 24 h, but reduced *IL-1β* expression at 48 h (0.55 ± 0.1-fold lower than MAP; *p* < 0.05, *n* = 3) and dramatically decreased its expression at 72 h (0.58 ± 0.04-fold decrease versus the MAP group, *p* < 0.01, *n* = 3) (Figure 6B). Post-treatment with MGO, *IL-6* was consistently suppressed at 24 h, 48 h, and 72 h, with a 0.66 ± 0.05-fold decrease (*p* < 0.01, *n* = 3); a 0.42 ± 0.05-fold decrease (*p* < 0.0001, *n* = 3); 0.19 ± 0.03-fold decrease (*p* < 0.0001, *n* = 3), respectively (Figure 6C). The remarkable reduction in *IL-6* was observed at 48 h and 72 h. In parallel, the rapidly expressed inflammatory cytokine *TNF-α* showed a significant reduction in its levels after 24 h of MGO treatment compared with MAP (Figure 3).

### 2.7. MGO Exhibits Antioxidant Properties in MAP-Infected THP-1 Macrophages by Associating Nrf-2/HO-1 Antioxidant Pathway

MGO may mitigate inflammation either by activating the nuclear factor erythroid 2-related factor/heme oxygenase-1 (Nrf-2/HO-1) antioxidant pathway and/or by reducing lactate export and downregulating MCT-4 and glycolytic signaling. This may occur via suppression of HIF-1α-associated downstream targets, including *GLUT-1*, *IL-1β*, *PDK1*, and PDH, which consequently weaken pro-inflammatory signaling and attenuate the lactate-associated pro-inflammatory marker profile in MAP-infected macrophages.

**Figure 7:**
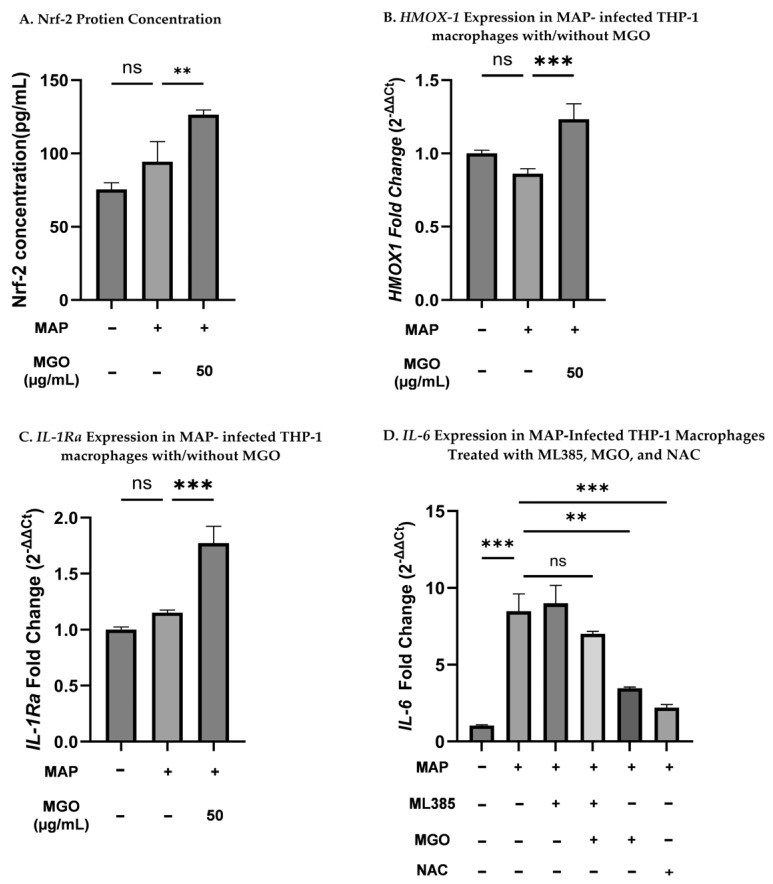
MGO attenuates MAP-induced inflammatory activation in THP-1 macrophages in association with enhanced Nrf-2 antioxidant responses. THP-1 cells were differentiated with PMA, infected with MAP, and then treated with 50 µg/mL MGO for 24 h. The supernatant and RNA were subsequently collected. (**A**) Nrf-2 protein concentration. (**B**) RT-qPCR expression of *HMOX1*. (**C**) *IL-1Ra* (*IL-1RN*) gene expression was calculated by the 2^−ΔΔCt^ method for the MAP infection and the MGO-treated group. (**D**) *IL-6* mRNA expression in MAP-infected THP-1 macrophages treated with ML385, MGO, or NAC. Data represent mean  ±  SEM; significance was assessed using one-way ANOVA with a post hoc Bonferroni correction. Data shown for at least *n* = 3. ** *p* < 0.01, *** *p* < 0.001. ns: not statistically significant.

To investigate whether Nrf-2-associated antioxidant signaling contributes to the cellular response to MGO treatment, Nrf-2 protein concentration and the expression of the Nrf-2-associated genes *HMOX1* and *IL1RN* were evaluated. Nrf-2 concentration was measured in the supernatants of polarized THP-1 macrophages after 24 h of MAP infection and a subsequent 24 h exposure to 50 µg/mL MGO. The antioxidant capacity of MGO was also assessed via the Nrf-2 downstream signal, such as *HMOX-1* expression level and the anti-inflammatory *IL-1RN*. Interestingly, Nrf-2 levels were significantly elevated after treatment with 50 µg/mL MGO compared with MAP-infected macrophages (1.34 ± 0.02-fold upregulation versus MAP; *p* < 0.01, *n* = 3; Figure 7A). Moreover, the target antioxidant transcription genes, *HMOX-1* and the anti-inflammatory *IL-1Ra* (*IL-1RN*), were markedly upregulated after 24 h of MGO exposure, with 1.43 ± 0.07-fold increase and 1.54 ± 0.08-fold increase, respectively (Figure 7B,C; *p* < 0.001, *n* = 3). The results indicate activation of Nrf-2-associated antioxidant and anti-inflammatory response.

Previous mechanistic studies in macrophages demonstrated that Nrf-2 can occupy regulatory regions proximal to the *IL-6* locus and inhibit inflammatory *IL-6* transcription by limiting RNA polymerase II recruitment. This transcriptional repression was reported to occur independently of the canonical antioxidant response element motif and was also observed in human THP-1 cells following pharmacological activation of Nrf-2 [44]. To further evaluate whether the antioxidant Nrf-2-associated signaling contributes to the anti-inflammatory effect of MGO, MAP-infected THP-1 macrophages were treated with ML385 prior to MGO exposure, and *IL-6* expression was measured as a downstream inflammatory readout (Figure 7D). ML385 is a small-molecule Nrf-2 inhibitor that binds to the Neh1 CNC-bZIP domain of Nrf-2 and interferes with the binding of the Nrf-2–MAFG complex to regulatory DNA, thereby suppressing Nrf-2-dependent transcriptional activity. MAP infection markedly increased *IL-6* expression compared with the uninfected control group (8.48 ± 0.79-fold versus control; *p* < 0.001; *n* = 3), confirming robust pro-inflammatory activation in THP-1 macrophages. Treatment with ML385 did not significantly alter MAP-induced *IL-6* expression (1.08 ± 0.20-fold versus MAP; ns; *n* = 3). In contrast, MGO treatment significantly reduced *IL-6* expression in MAP-infected macrophages. However, this reduction was attenuated in the presence of ML385, whereas *IL-6* expression in the MAP + ML385 + MGO group was approximately 2.02 ± 0.04-fold higher than in the MAP + MGO group (*p* < 0.05; *n* = 3). These findings indicate that blocking Nrf-2 with ML385 weakened the ability of MGO to reduce *IL-6* expression, supporting a contribution of Nrf-2-associated signaling to the MGO-mediated anti-inflammatory response. In addition, the results support the involvement of Nrf-2-associated signaling in the anti-inflammatory activity of MGO but do not establish complete or exclusive Nrf-2 dependence. Therefore, additional mechanisms, including Nrf-2-independent antioxidant responses and modulation of metabolic or lactate-associated inflammatory signaling, may also participate.

NAC, used as an antioxidant positive control, produced a greater reduction in *IL-6* expression (0.26 ± 0.04-fold versus MAP; *p* < 0.001; *n* = 3). Importantly, the MGO-treated group showed a reduction in *IL-6* levels, consistent with NAC and supporting the antioxidant-sensitive anti-inflammatory activity of MGO in MAP-infected THP-1 macrophages.

### 2.8. MGO Suppresses Glycolysis/Lactate-Associated Marker Expression in MAP-Infected THP-1 Macrophages

To evaluate whether MGO alters lactate/MCT4-associated inflammatory markers in MAP-infected THP-1 macrophages, we examined RNA expression of MCT4, encoded by *SLC16A3*, measured extracellular lactate export, and evaluated selected glycolysis-/lactate-associated markers, including GLUT1 (*SLC2A1*), *LDHB*, *EGLN1*/PHD2, *HIF-1α*, *PDK1*, *PDHA1*, and *IL-1β*, after 24 h of MGO treatment. We also sought to explore how PHD2 affects HIF-1α expression levels following MGO treatment. Last but not least, we investigated how downstream HIF-1α signaling pathways, including *PDK-1*, *PDHA1*, *LDH-B*, *IL-1β*, and *GLUT-1*, can dampen the lactate-related inflammatory profile induced by MAP infection in THP-1 macrophages.

**Figure 8:**
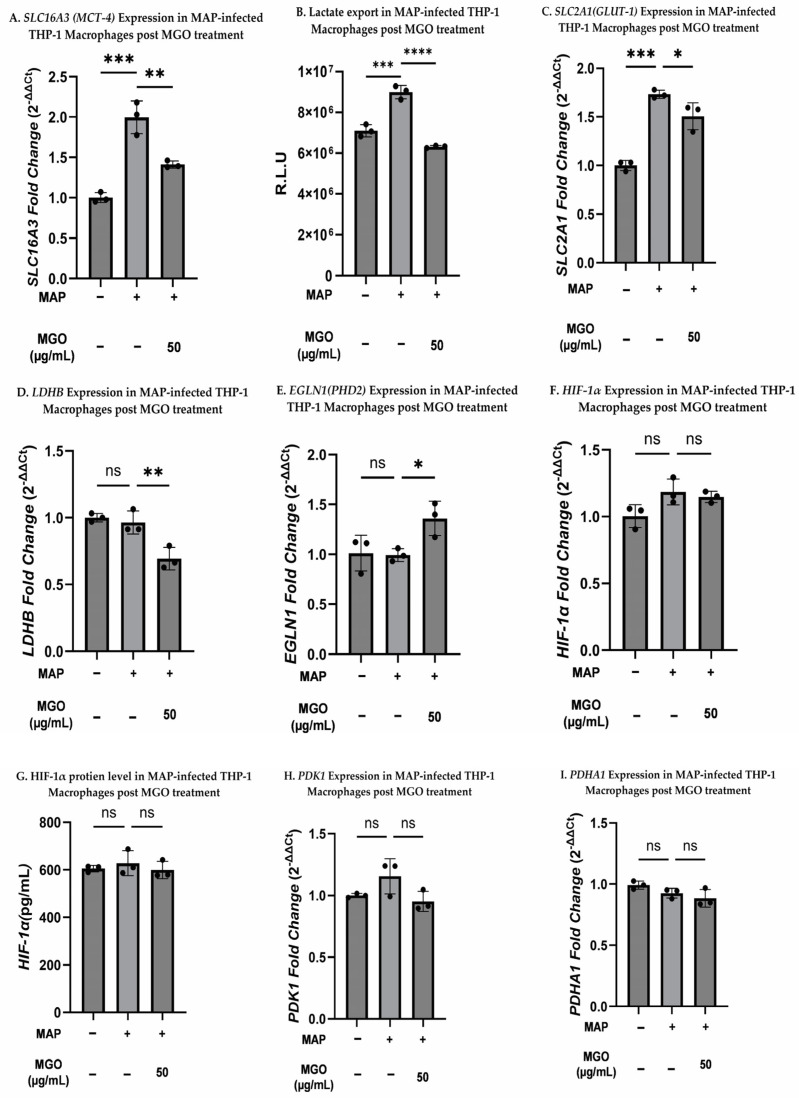
MGO attenuates MAP-induced lactate export and expression of glycolysis-associated inflammatory markers in THP-1 macrophages. MGO downregulates MCT-4 expression and lessens lactate export in MAP-infected THP-1 macrophages. (**A**) THP-1 cells were differentiated with PMA, followed by infection with MAP for 24 h, and then incubated with 50 µg/mL MGO for 24 h. Infected and uninfected THP-1 macrophages were subjected to RNA extraction followed by RT-qPCR for *SLC16A3* expression levels. (**B**) THP-1 cells were differentiated and plated in dialyzed FBS medium, followed by MAP infection, and then treated with 50 µg/mL MGO for 24 h. Lactate in the supernatant was measured by luminescence assay, and results are expressed as Relative Luminescence Units (R.L.U.). (**C**) represents the gene expression of *SLC2A1* (calculated with the 2^−ΔΔCt^ method) in response to MAP infection and MGO treatment. (**D**) represents the gene expression of *LDHB* (calculated with the 2^−ΔΔCt^ method) in response to MAP infection and MGO treatment. (**E**) represents the expression levels of *EGLN1* in MAP-infected THP-1 macrophages post MGO treatment. Then, HIF-1α expression was examined at the mRNA and protein levels. (**F**) represents RT-qPCR expression of HIF-1α calculated with the 2^−ΔΔCt^ method. (**G**) represents HIF-1α protein concentration (determined by ELISA). (**H**) represents the gene expression of *PDK1* following MGO treatment for MAP- infected THP-1 macrophages (calculated with the 2^−ΔΔCt^ method). (**I**) represents *PDHA1* gene expression in THP-1 cells following MAP infection and MGO treatment. Each group has three biological replicates. The data are presented as mean ± SEM; *n* = 3. * *p* < 0.05, ** *p* < 0.01, *** *p* < 0.001, **** *p* < 0.0001 are considered statistically significant. ns: not statistically significant.

The results demonstrated that MGO significantly reduced *SLC16A3* expression relative to the MAP-infected group (0.71 ± 0.01-fold lower than MAP; *p* < 0.001; Figure 8A). Accordingly, *MCT4* downregulation was accompanied by a marked decrease in lactate export in MGO-treated cells relative to MAP infection alone (Figure 8B; *p* < 0.0001; 0.7-fold decrease versus MAP). Collectively, MGO dampens glycolysis in MAP-infected THP-1 cells via downregulation of GLUT1 by 0.87 ± 0.05-fold versus the MAP-infected group (Figure 8C; *p* < 0.05, *n* = 3). Treatment with MGO resulted in a significant reduction in *LDHB* expression relative to MAP alone (0.72 ± 0.05-fold lower than the MAP group; *p* < 0.01; Figure 8D), consistent with a shift away from LDH-associated glycolytic flux and/or reduced lactate-linked metabolic activity in response to MGO.

Next, we interrogated whether reduced extracellular lactate levels were associated with changes in EGLN1/PHD2 expression and HIF-1α-associated downstream markers. The results illustrated that *EGLN1* gene expression levels were increased in the MGO-treated group (*p* < 0.05, *n* = 3), with a 1.37 ± 0.10-fold increase compared with MAP-infected THP-1 macrophages (Figure 8E). In THP-1 macrophages, 6 h of MAP exposure produced a marked change in HIF-1α transcriptional levels, while adding 50 µg/mL MGO did not alter HIF-1α transcript levels after 6 h, 24 h, or 48 h of treatment. However, there was no significant difference in *HIF-1α* mRNA or protein levels among the tested groups when THP-1 macrophages were infected with MAP for 24 h, followed by 24 h of MGO treatment (Figure 8F,G). *PDK1* gene expression levels were moderately reduced by 0.82 ± 0.04-fold relative to MAP alone (*n* = 3; Figure 8H), whereas PDHA1 expression levels were not significantly changed among all the tested groups (*n* = 3; Figure 8I). Therefore, these data showed that MGO can attenuate lactate-associated inflammatory signaling.

### 2.9. MGO Attenuates MAP-Induced TNF-α Expression and Shows Overlap with an α-CHC-Sensitive MCT/Lactate-Linked Pathway

**Figure 9:**
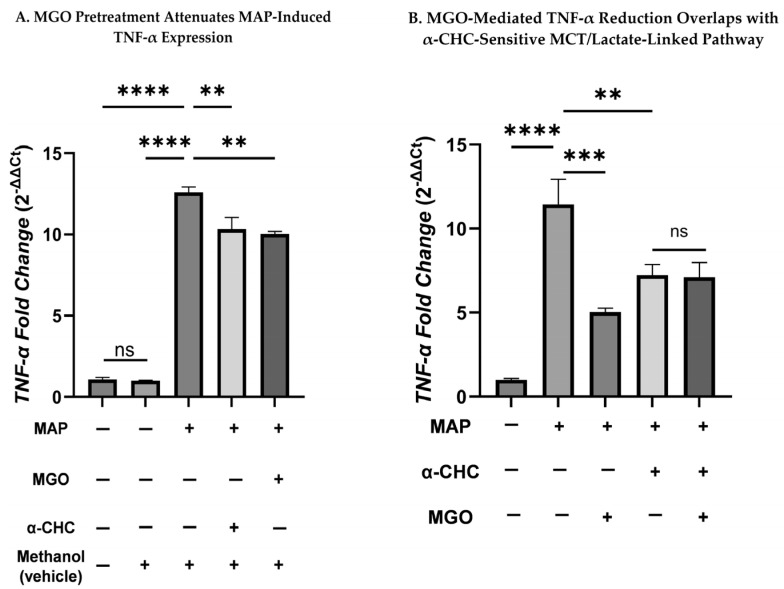
MGO attenuates MAP-induced *TNF-α* expression and displays convergence on the α-CHC-sensitive MCT/lactate-linked inflammatory pathway. (**A**) PMA-differentiated THP-1 macrophages were pretreated with either α-CHC (0.25 mM) or MGO (50 µg/mL) for 3 h before MAP. At 24 h post-infection, cells were lysed for RNA extraction, and *TNF-α* expression was measured by RT-qPCR. Methanol was included as the vehicle control for α-CHC treatment. (**B**) MAP-infected THP-1 macrophages were treated with α-CHC (0.25 mM) for 3 h, then with/without MGO (50 µg/mL) for 24 h. *TNF-α* expression was calculated using the 2^−ΔΔCt^ method. Data are presented as mean ± SEM; *n* = 3. Statistical significance was determined by one-way ANOVA with Bonferroni multiple-comparison correction. ns, not significant; ** *p* < 0.01, *** *p* < 0.001, **** *p* < 0.0001. ns: not statistically significant.

To evaluate whether MGO modulates MAP-induced inflammatory activation through an MCT/lactate-linked mechanism, two complementary experimental approaches were performed. First, THP-1 macrophages were pretreated with either 0.25 mM α-CHC or 50 µg/mL MGO for 3 h before MAP infection to determine whether MGO confers a protective anti-inflammatory effect against subsequent MAP challenge. MAP infection markedly increased *TNF-α* mRNA expression compared with uninfected control cells (Figure 9A; 12.60 ± 0.23-fold; *p* < 0.0001; *n* = 3). Pretreatment with α-CHC significantly reduced MAP-induced *TNF-α* expression to 0.82 ± 0.04-fold relative to MAP (*p* < 0.01; *n* = 3), suggesting that monocarboxylate transporter activity contributes to MAP-associated inflammatory signaling. Similarly, pretreatment with 50 µg/mL MGO significantly reduced TNF-α expression compared with MAP-infected cells (Figure 9A; 0.80 ± 0.09-fold decrease vs. MAP; *p* < 0.01; *n* = 3). The significant reduction observed after MGO pretreatment supports an anti-inflammatory protective effect of MGO in this model.

Second, to investigate whether the anti-inflammatory effect of MGO is associated with the MCT/lactate pathway, MAP-infected THP-1 macrophages were treated with 0.25 mM α-CHC for 3 h before MGO treatment. After 24 h of MGO treatment, *TNF-α* expression, a downstream readout for MCT/lactate, was measured by RT-qPCR (Figure 9B). MAP infection markedly increased TNF-α expression relative to the uninfected control group (11.44 ± 0.87-fold; *p* < 0.0001; *n* = 3). Treatment with MGO significantly reduced MAP-induced TNF-α expression compared with MAP (Figure 9B; *p* < 0.001; *n* = 3). Also, α-CHC pretreatment reduced TNF-α expression to 0.63 ± 0.06-fold relative to MAP (*p* < 0.01; *n* = 3). However, when MGO was added after α-CHC pretreatment, *TNF-α* expression was not further reduced compared with α-CHC alone, with similar levels observed in the MAP + CHC + MGO and MAP + CHC groups (0.62 ± 0.07-fold versus MAP and 0.63 ± 0.06-fold versus MAP, respectively; ns), suggesting that the *TNF-α* downregulating effect of MGO may overlap with or converge on the α-CHC-sensitive MCT/lactate-linked inflammatory pathway.

### 2.10. Hormetic Dosage of MGO Did Not Induce MG-H1 Protein Adduct Diabetic Levels After MGO Supplementation in THP-1 Macrophage Following MAP Infection

**Figure 10:**
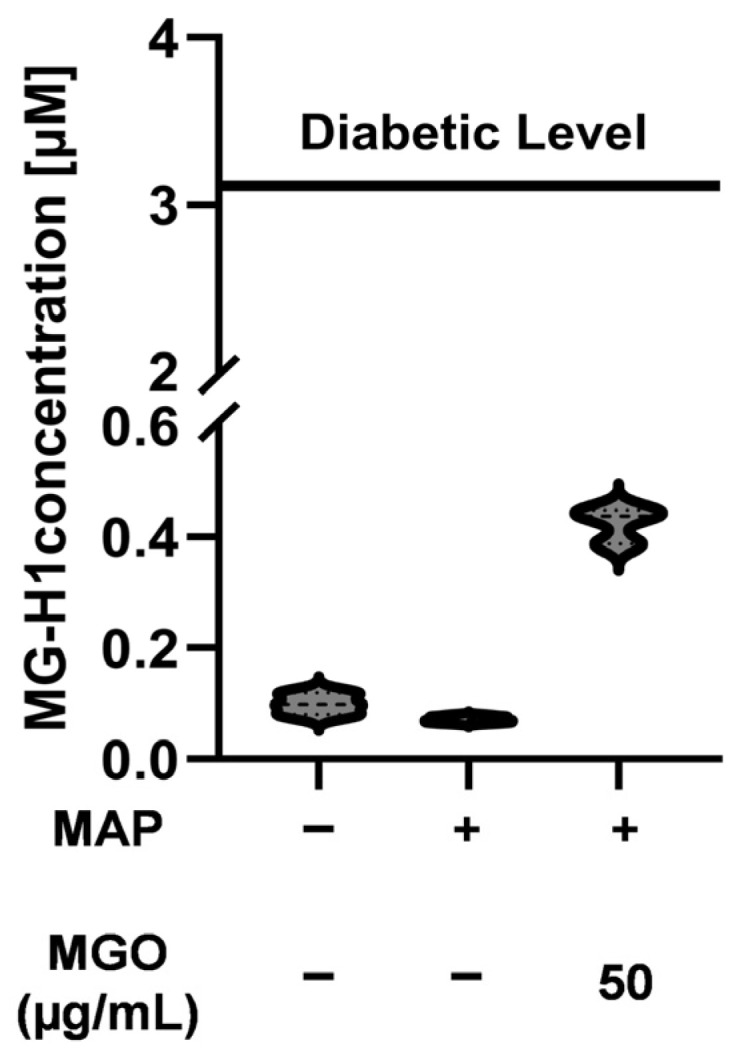
MG-H1 concentration in MAP-infected THP-1 macrophages post MGO treatment. THP-1 cells were differentiated with PMA, followed by infection with MAP for 24 h, and then incubated with 50 µg/mL MGO for 24 h. Infected and uninfected THP-1 macrophages were subjected to quantification of MG-H1 using the OxiSelect™ MGO Competitive ELISA Kit (Cat. No. STA-811), which was obtained from Cell Biolabs, Inc., San Diego, CA, USA. Each group contains three biological replicates (*n* = 3).

MGO reacts with protein, nucleotides, and basic phospholipids in a process called glycation, and the formed adducts are called advanced glycation end products (AGEs) [41]. The most abundant quantitative and functional AGEs in physiological systems are arginine-derived hydroimidazolones (MG-H1) [42]. MG-H1 levels were quantified in MAP-infected THP-1 macrophages following MGO supplementation. As shown in Figure 10, control THP-1 cells produced MG-H1 levels, while MAP infection did not significantly alter MG-H1 levels relative to the control group. Following MGO supplementation, MG-H1 levels record a modest rise compared with both the control and MAP groups. However, even after MGO treatment, MG-H1 concentrations remain well below the diabetes threshold (3 µM). Importantly, these data demonstrate that the MGO dose used in this study does not induce pathological carbonyl stress or sustained protein glycation characteristic of diabetic or hyperglycemic states.

## 3. Discussion

Although substantial research efforts have been directed toward CD, it remains a chronic, debilitating disorder without a definitive cure [45]. Anti-*TNFα* monoclonal antibodies, including infliximab and adalimumab, remain central therapeutic options for moderate-to-severe CD [46]; however, their long-term effectiveness is limited by primary non-response, secondary loss of response, immunogenicity, and increased susceptibility to infections, including mycobacterial infections [47,48]. Moreover, experimental evidence suggests that *TNFα* blockade may create favorable conditions for the survival of MAP, particularly in MAP-positive CD patients [13]. In parallel, co-cultures of THP-1 with Caco-2/HT-29 demonstrated that neutralizing the *IL-6* receptor during MAP challenge intensified barrier injury [14]. Therefore, there is a high demand for alternative, affordable approaches to managing inflammation while preserving, or ideally enhancing, host antimicrobial competence in MAP-positive CD patients. MGO is an essential and integral constituent of Manuka honey, which is known to be beneficial in the treatment of many diseases [33,34,35]. In this study, MGO clearly demonstrated antibacterial activity (Figure 1C), chemical stability, and sustained activity against MAP. Despite prolonged preincubation of MGO in lab culture media, MAP growth remained markedly restricted at >80% in the presence of MGO, even after several weeks of incubation (Figure 1D). This study demonstrated that MGO modulates M1/M2-like phenotype polarization, inflammatory cytokine production, and lactate-associated inflammatory markers in MAP-infected THP-1 macrophages. These findings support a role for MGO in attenuating MAP-induced inflammatory activation. It is noteworthy that this study is the first to report that MGO, when administered at hormetic doses, acts as an antioxidant- and anti-inflammatory-associated metabolite and as a modulator of glycolysis- and lactate-linked inflammatory markers in in vitro MAP-associated CD-like macrophage model.

It is speculated that 0.1% to 0.4% of MGO production originates from the glycolytic flux [32] and that infected and trained immune cells exhibit a high rate of aerobic glycolysis [49]. In this study, MAP-infected macrophages produce MGO endogenously at levels higher than in controls, but not sufficient to neutralize MAP infection (Figure 1A). The current results show that supplying MGO exogenously can act as an innate antibacterial effector and diminish MAP growth. Additionally, the data show that 25–50 µg/mL of 40% MGO did not compromise macrophage viability; thus, changes in secreted mediators reflect immunomodulation rather than cell loss. In addition, 50 µg/mL MGO showed no appreciable cytotoxicity at 24 h, as LDH release was unchanged and cell viability remained at 90–96%. These results indicate a promising safety window and justify its use in subsequent experiments. Consistent with the study findings, a previous research study showed that mice given MGO orally over a long period of time exhibited a mild increase in plasma MGO; nevertheless, they lived longer, had fewer age-related solid tumors, and did not develop diabetes or kidney dysfunction [38]. The aforementioned findings support further preclinical evaluation of MGO as a feasible, low-cost therapeutic candidate and an anti-inflammatory modulator in MAP-associated CD models. MAP-infected THP-1 macrophages exhibit M1-like morphology (Figure 4D) and, concurrently, show increased production of pro-inflammatory mediators. In particular, *CXCL10*, a chemokine that serves as an M1 marker, is elevated (Figure 4G) during M1 polarization phenotype, and the pro-inflammatory cytokines *TNF-α*, *IL-1β*, and *IL-6* are significantly upregulated (Figure 3, Figure 6B and Figure 6C, respectively). Conversely, MGO treatment skews MAP-infected THP-1 macrophages away from M1 polarization toward an M2-like phenotype, with a more uniform, less branched morphology. We observed that most cells adopted a rounded morphology consistent with M2 polarization, indicating cytoskeletal remodeling. MGO strikingly downregulated *CXCL10* and significantly reduced the pro-inflammatory cytokines *TNF-α*, *IL-1β*, and *IL-6*, thereby shifting cells away from the M1-like program. Consistent with this transition, MGO upregulated CD206, a canonical M2 marker, and markedly elevated *IL-10*, reinforcing an anti-inflammatory phenotype in MAP-infected THP-1 macrophages. Furthermore, MGO treatment significantly downregulated *TNF-α* levels, even when administered prior to MAP infection. Overall, these findings support the conclusion that a low-hormetic dose of MGO tempers macrophage inflammatory programming in MAP-positive CD, thereby bolstering its potential as an adjunct strategy to rebalance host responses without compromising cell viability.

The mechanism by which MGO acts in CD tissue is unclear. In this regard, we assessed the impact of an exogenous MGO augment dose on the Nrf-2 antioxidant pathway in MAP-infected macrophages. Nrf-2 is a stress-responsive transcription factor that protects cells from oxidative injury [50]. When Nrf-2 accumulates in the nucleus, it reinforces the antioxidant program and tempers inflammatory signaling. Among the induced genes are heme oxygenase-1 (*HMOX-1*), reduced glutathione (GSH), and the anti-inflammatory Interleukin-1 receptor antagonist (*IL-1Ra*) [50]. In this study, MGO administration showed activation of the Nrf-2 pathway, as demonstrated by an elevated concentration of Nrf-2 protein (1.4-fold) in MGO-treated MAP-infected macrophages. Subsequently, Nrf-2 translocated to the nucleus and bound to its coactivator CBP/P300, thereby remarkably upregulating the transcription of the antioxidant gene *HMOX-1 (HO-1)* and the anti-inflammatory *IL-1Ra* (Figure 7A–C). *HO-1* can exert an anti-inflammatory effect by suppressing p65 activation, thereby dampening NF-κB signaling [51]. Consistent with these data, our results demonstrated that MGO treatment activates Nrf-2, which, in turn, upregulates *HO-1* and *IL-10* and inhibits NF-κB downstream signaling, including *TNF-α*, *IL-1β*, and *IL-6*, in MAP-infected THP-1 macrophages. Therefore, activating the Nrf-2/*HO-1* axis by augmenting an exogenous dose of MGO not only acts as an antioxidant but also exhibits anti-inflammatory properties. In addition, MGO may activate the defense system against glycation, thereby preventing protein damage via the KEAP1/Nrf-2 pathway. Thus, there is an induction of antioxidant enzymes, which are involved in cellular protection against glycation. Since MGO influences multiple strategies, including anti-bacterial activity and enhancement of antioxidant and anti-inflammatory signaling pathways, it supports further preclinical investigation in MAP-associated CD models.

The ML385/NAC experiment further supports the involvement of antioxidant-associated signaling in the anti-inflammatory effect of MGO. MGO markedly reduced MAP-induced *IL-6* expression; this reduction was moderately attenuated by the Nrf-2 inhibitor ML385, suggesting that Nrf-2-associated signaling contributes to MGO downregulation of *IL-6* expression. In parallel, NAC, used as an antioxidant positive-control comparator, strongly reduced MAP-induced *IL-6* expression, supporting an antioxidant-sensitive component in the inflammatory response of MAP-infected THP-1 macrophages (Figure 7D). Additionally, the present study demonstrated that ML385 alone did not significantly alter MAP-induced *IL-6* expression; however, its addition significantly attenuated the reduction in *IL-6* produced by MGO. This comparison suggests that the anti-inflammatory effect of MGO contains Nrf-2-sensitive components. Consistent with this interpretation, previous work demonstrated that Nrf-2 can occupy regulatory regions proximal to the IL-6 locus in macrophages and inhibit inflammatory transcription by limiting RNA polymerase II recruitment [44]. Nevertheless, the pharmacological inhibition with ML385 did not completely restore *IL-6* expression to the MAP-infected level. Overall, these findings support a partial role for Nrf2-associated signaling in the anti-inflammatory response to MGO, while also indicating that the suppression of *IL-6* likely involves additional antioxidant and metabolic mechanisms.

MCT4 is the leading H+/lactate exporter in highly glycolytic cells [52,53]. MCT4 is mechanistically upregulated under hypoxia through the binding of HIF-1α to two hypoxic response elements (HRE) within the *SLC16A3* promoter [54,55]. Lactate is considered an end product of aerobic glycolysis, in which pyruvate is converted to lactate by the lactate dehydrogenase enzyme (LDH) [56]. Our results revealed that MAP-infected THP-1 macrophages significantly upregulated MCT-4, increased lactate export (as a glycolysis fuel), and upregulated GLUT-1 (Figure 8A–C), confirming the glycolytic shift. GLUT1 is highly expressed in infected macrophages to support glucose uptake and sustain glycolysis. Our data indicate that MGO acts as a metabolic brake during M1 polarization of MAP-infected THP-1 cells by reducing lactate export through the downregulation of MCT-4 (Figure 8A,B). MGO also attenuates the glycolysis-associated pro-inflammatory state by downregulating GLUT1 expression levels in MAP-infected macrophages (Figure 8C). Moreover, MGO supplementation to the MAP-THP-1-infected group resulted in a significant reduction in *LDH-B* expression relative to MAP alone (Figure 8D). Collectively, these findings support a shift in metabolic balance during MAP infection in the presence of MGO away from lactate-driven pro-inflammatory metabolism. These findings are consistent with reduced lactate-associated inflammatory signaling; however, direct metabolic flux and mitochondrial respiration remain to be tested.

It has been reported that lactate can robustly activate inflammatory macrophages by directly binding to the catalytic domain of PHD2 in a competitive manner with α-ketoglutarate, thereby stabilizing HIF-1α [29]. In fact, factor inhibiting HIF (FIH) hydroxylates a conserved asparagine residue in HIF-1α, thereby blocking recruitment of coactivators such as p300/CBP and limiting transactivation [57,58]. The roles of PHDs and FIH in regulating HIF-1α stability and coactivator engagement are well established under hypoxic conditions. As a result, increased lactate levels in inflammatory macrophages exacerbate the pro-inflammatory environment and promote chronic inflammation in infected macrophages. On the other hand, elevated lactate levels in M0 macrophages shift macrophages towards M2-like polarization [29]. Considering these reports, we found that applying an MGO dose to MAP-infected macrophages reduced cytosolic lactate levels, thereby relieving lactate-driven suppression of PHD2 activity, consistent with the observed increase in *Egln1*(PHD2) (Figure 8E). Logically, *Egln1* upregulation was expected to lower HIF-1α stability as part of the HIF-negative feedback loop. However, our results showed no statistically significant differences in *HIF-1α* mRNA or protein levels across all treated groups (Figure 8F,G). Interestingly, when MGO was applied to MAP-infected macrophages, *HIF-1α* downstream targets, such as GLUT1 and *IL-1β*, were downregulated. As expected, PDK1 has also been shown to be downregulated after MGO augmentation (Figure 8H). Nevertheless, *PDHA1* mRNA expression levels showed no statistically significant differences among the groups tested (Figure 8I). Further investigation is needed to assess the level of phosphorylated PDH protein. There are several potential scenarios that may explain why our HIF-1α results showed no statistical significance at the mRNA and protein levels in the MAP-infected and MGO-treated macrophage groups (Figure 8F,G). Previous research in the hypoxia field has emphasized the vital role of HIF-1α’s transactivation capacity, particularly its ability to recruit coactivators, such as p300/CBP [59,60]. FIH is a key enzyme that regulates the transactivation capacity of HIF-1α. By hydroxylating an asparagine residue in HIF-1α, FIH blocks the recruitment of co-activators [61]. Clearly, treating MAP-infected macrophages with MGO upregulated PHD2 expression, as measured by *Egln1* mRNA levels (Figure 8E). This suggests that MGO also elevates FIH activity, consequently blocking HIF-1α function by inhibiting co-activator recruitment. As a result, we observe that treating MAP-infected macrophages with MGO decreases the expression levels of downstream HIF-1α signaling targets, *GLUT1*, *IL-1β*, and *PKD1*. Although *HIF-1α* mRNA and protein levels were not significantly altered following MGO treatment, several HIF-1α-associated downstream targets showed decreased expression. This suggests that MGO may influence HIF-1α transcriptional activity rather than total HIF-1α abundance. One possible explanation is altered coactivator recruitment through FIH-mediated repression; however, other mechanisms may also contribute, including changes in HIF-1α hydroxylation status, protein stability, or post-translational regulation. Another possible explanation is the short half-life and rapid turnover of HIF-1α. Our results showed that MAP-THP-1 macrophages, when cultured for 6 h, exhibited a marked increase in HIF-1α transcript levels. However, treatment with 50 µg/mL MGO did not significantly alter *HIF-1α* mRNA expression at 6, 24, or 48 h compared with MAP-infected cells. This suggests that the effect of MGO on HIF-1α-related signaling may occur through post-translational regulation.

MGO also enhances the Nrf-2 detoxification machinery by upregulating HO-1 and other antioxidant genes. As a result, cells clear ROS more efficiently, and this shift in redox tone is the primary reason for the decrease in HIF-1α activity. This Nrf-2–HIF-1α interaction is consistent with established redox biology. ROS can help stabilize HIF-1α by limiting prolyl hydroxylase activity, which normally tags HIF-1α for degradation. In addition, Nrf-2 promotes greater antioxidant capacity and a metabolic state less supportive of inflammatory glycolysis. Nrf-2 behaves like a “fire suppressant” as it is triggered by an initial oxidative spark, but then it prevents the later ROS-dependent reinforcement of HIF-1α-driven immunometabolic activation. Collectively, in our model, MGO shifts MAP-infected macrophages into an Nrf-2-dominant state, and this change propagates through the inflammatory state, redox balance, and PHD2/HIF-1α signaling. Moreover, when MGO activates Nrf-2, it allows Nrf-2 to accumulate in the nucleus and drive an antioxidant transcriptional program and anti-inflammatory signals. Consequently, Nrf-2 blunts NF-κB-linked inflammatory cytokines (*TNF-α*, *IL-1β*, *IL-6*), thereby dampening them, while anti-inflammatory mediators (including *IL-10* and *IL-1Ra*) rise in parallel. Nrf-2 also promotes the transcription of antioxidant defenses, including *HMOX1* and related cytoprotective enzymes, thereby reducing intracellular ROS and oxidative stress. In parallel, our data indicate that MGO suppresses glycolytic drive by downregulating GLUT1 (*SLC2A1*) and reducing lactate export via MCT4 (*SLC16A3*) downregulation, resulting in lower extracellular lactate. Lactate is known to stabilize HIF-1α in multiple contexts, in part by inhibiting PHD2 activity (via lactate/α-KG competition), so reducing lactate pressure would be expected to restore PHD2 function, consistent with our finding that MAP-infected macrophages with MGO upregulated PHD2 expression, as observed by *Egln1* mRNA levels. This suggests that MGO also elevates FIH activity, thereby blocking HIF-1α function by inhibiting CBP/P300 co-activator recruitment as a post-transcriptional modification. As a result, we observed that MGO decreases the expression levels of downstream HIF-1α signaling targets, *GLUT1*, *IL-1β*, and *PKD1*. Putting these layers together explains what we observe experimentally: MGO treatment mitigates MAP-driven inflammatory activation in THP-1 macrophages by promoting antioxidant response signaling and limiting the expression of lactate-associated pro-inflammatory markers.

The data imply that MGO acts as a protective agent against MAP-induced inflammation by downregulating *TNF-α* when supplemented prior to MAP infection (Figure 9A). Consistent with the lactate/MCT-associated inflammatory model, α-CHC pretreatment reduced MAP-induced *TNF-α* expression, supporting the contribution of monocarboxylate transporter activity to MAP-driven macrophage inflammation. Furthermore, administration of MGO after α-CHC pretreatment in MAP-infected macrophages did not yield an additional reduction in TNF-α compared with α-CHC alone, suggesting that the *TNF-α* downregulation effect of MGO may overlap with or converge on the α-CHC-sensitive MCT/lactate-linked inflammatory pathway. These findings support the involvement of lactate-associated signaling in the anti-inflammatory effect of MGO (Figure 9B).

Indeed, MGO modifies nucleophilic targets, including guanine residues in DNA and amino acid side chains such as arginine, lysine, and cysteine, thereby promoting the formation of advanced glycation end products (AGEs) [62], which may lead to diabetes. In this study, the hormetic dosage of MGO treatment resulted in a moderately elevated MG-H1 without reaching pathological AGE levels (Figure 10), as the threshold for AGEs in diabetes is above 3 µM [63,64], indicating that the concentration used in this study is safe for patients.

## 4. Materials and Methods

### 4.1. THP-1 Macrophages Cell Culture

THP-1 monocytes were obtained from American Type Culture Collection (ATCC; Cat# TIB-202, Manassas, VA, USA) and were cultured in RPMI-1640 high-glucose medium containing L-glutamine and HEPES (ATCC, Cat. No. 30-2001, Manassas, VA, USA), supplemented with 10% fetal bovine serum (FBS; Sigma Life Science, St. Louis, MO, USA) and 0.005 mM of 2-Mercaptoethanol (Cat# 31350010, Gibco, Thermo Fisher Scientific). The cells were grown to 80% confluency in T-75 cell culture flasks (Cat# 12-565-349, Thermo Fisher Scientific, Nunc, Waltham, MA, USA) for 48 h at 37 °C in a humidified 5% CO_2_ incubator. A total of 5 × 10^5^ THP-1 monocytes were plated in 12-well tissue culture plates and differentiated into macrophages using 50 ng/mL phorbol 12-myristate 13-acetate (PMA; Cat# P8139-1MG, Sigma Life Science, St. Louis, MO, USA) for 48 h.

### 4.2. Evaluating MGO Effect on MAP Growth in MGIT Media

MAP clinical isolate UCF4 was cultured in modified Middlebrook 7H9 broth-based media supplemented with BACTEC MGIT ParaTB Supplement (Cat# 245156, Becton, Dickinson and Company, Franklin Lakes, NJ, USA) and ferric mycobactin J (Allied Monitor, Inc., Fayette, MO, USA; Cat. 62-0002) in the BACTEC MGIT 320 instrument (Becton, Dickinson and Company). Culture was maintained in the BACTEC MGIT 320 system (BD) using MGIT ParaTB medium tubes (Cat. #245154, Becton, Dickinson and Company) with an integrated fluorescent oxygen sensor (BD, Cat. #245154). The indicator is embedded in silicone at the tube base, and dissolved oxygen quenches fluorescence. As MAP grows and consumes oxygen, quenching diminishes and fluorescence increases; the instrument converts these changes to growth units (GU) using internal calibration standards. All cultures were incubated under the manufacturer’s recommended conditions, and GU values were recorded at specified time points for downstream analysis. To investigate the antibacterial effect of MGO (40%, Sigma-Aldrich, St. Louis, MO, USA) on MAP, 50 μL of active MAP culture (CFU) was added to each tube supplemented with different concentrations of MGO (0, 5, 10, 15, 20, 25, and 50 μg/mL), and the MAP growth unit was monitored daily for 18 days. Percentage inhibition of bacterial growth was calculated from colony-forming units (CFU) as % Inhibition = [(CFU(control) − CFU(treated)/CFU (control)] × 100, where CFU (control) is the viable count of untreated cultures and CFU (treated) is the mean viable count of cultures exposed to the different concentrations of MGO. Next, to evaluate whether MGO remains active and maintains its protective antibacterial activity, we pretreated MGIT tubes supplemented with BACTEC MGIT ParaTB Supplement with MGO at 0, 50, and 100 μg/mL, then incubated them under standard conditions for 30 days before inoculating them with 50 μL of active MAP (UCF4). MAP growth was recorded over 24 days.

### 4.3. Effect of MGO on MAP in THP-1 Macrophages

MGO was purchased from Sigma-Aldrich (St. Louis, MO, USA) as a commercial aqueous MGO solution (~40% in H_2_O). Fresh working dilutions were prepared in sterile culture medium immediately before each experiment. All MGO concentrations reported refer to the final working concentration of MGO treatment prepared from the commercial aqueous stock solution. THP-1 macrophages were infected with MAP UCF4 at 1 × 10^7^ CFU/mL, corresponding approximately to a multiplicity of infection (MOI) of 20, for 24 h. This infection condition was selected based on optimized MAP–THP-1 macrophage protocols established in our laboratory to induce a reproducible MAP-associated inflammatory and metabolic response while maintaining sufficient macrophage viability for downstream analyses. Following the infection period, cells were washed with sterile PBS to remove extracellular/non-phagocytosed bacteria, and fresh culture medium was added before treatment. Cells were treated with the indicated final working concentrations of MGO for the specified time points. Cells were harvested for RNA extraction at the indicated time points after MGO treatment. In addition, to evaluate the direct effect of MGO on uninfected macrophages, THP-1 macrophages were treated with MGO alone at 25 µg/mL and 50 µg/mL. *TNF-α* expression was measured by qRT-PCR.

### 4.4. Quantitation of Endogenous MGO in MAP-Infected THP-1 Cells

THP-1 macrophages were seeded at 5 × 10^5^ cells/mL in a 12-well tissue culture plate using serum-free RPMI media for 48 h. Then, THP-1 macrophages were infected with MAP UCF4 (1 × 10^7^ CFU/mL) for 24 h. Supernatants and cell lysates from MAP-infected THP-1 macrophages and controls were then analyzed for MGO measurement using a competitive MGO ELISA kit (Cat# EKN53482-96T, Biomatik Corporation, Kitchener, ON, Canada) following the manufacturer’s procedure. Each experiment represents the average of three technical replicates.

### 4.5. Lactate Dehydrogenase (LDH)-Glo Cytotoxicity Assay

To evaluate whether MGO treatment induced cytotoxicity in MAP-infected THP-1 macrophages, cytotoxicity was assessed using the LDH-Glo™ Cytotoxicity Assay kit, Promega (Madison, WI, USA), according to the manufacturer’s instructions. This assay quantifies LDH released into the culture medium from membrane-damaged cells. In the LDH-Glo system, released LDH activity is coupled to a bioluminescent reaction, and the luminescent signal is proportional to the amount of LDH released. Briefly, THP-1 monocytes were resuspended in serum-free RPMI medium, differentiated with PMA, and seeded in 96-well opaque-sided plates at a density of 5 × 10^5^ cells/mL. Cells were incubated for 48 h to allow macrophage differentiation. After differentiation, THP-1 macrophages were washed, fresh medium was added, and then the macrophages were infected with MAP UCF4 at 1 × 10^7^ CFU/mL for 24 h. Following infection, cells were treated with the indicated concentrations of MGO for the specified time points. Luminescence was measured using a Promega™ GloMax Navigator GM-2000 plate reader (Madison, WI, USA) after 60 min of incubation at room temperature. Percent cytotoxicity was calculated relative to the maximum LDH release control generated with 10% Triton X-100, with appropriate spontaneous and maximum LDH release controls included per the manufacturer’s protocol. The percentage of cell cytotoxicity can be calculated using the following formula:

$$Percent\;Cytotoxicity=100\times \frac{Experimental\;LDH\;Release-Medium\;Background}{Maximum\;LDH\;Release\;Control-Medium\;Background}$$

### 4.6. Trypan Blue Exclusion Assay

Trypan Blue exclusion was used to assess the cell viability of uninfected THP-1 macrophages, MAP-infected THP-1 macrophages, and MAP-infected THP-1 macrophages exposed to different MGO concentrations for 24 h. Briefly, THP-1 macrophages were infected with MAP UCF4 (1 × 10^7^ CFU/mL) for 24 h, followed by treatment with different concentrations of MGO for 24 h. Equal volumes of cell suspension and Trypan Blue stain 0.4% (Cat#T10282, Invitrogen, Thermo Fisher Scientific) were mixed, then transferred to a Countess cell counting chamber slide (Cat# C10283, Invitrogen, Thermo Fisher Scientific) and counted using a Countess 3 FL automated cell counter (Invitrogen, Thermo Fisher Scientific).

### 4.7. RNA Extraction and RT-qPCR

RNA was extracted using the RNeasy Mini Kit (Qiagen, Hilden, Germany), and RNA concentration was quantified using a NanoDrop OneC Microvolume UV-Vis Spectrophotometer (ThermoFisher Scientific, Waltham, MA, USA). RNA was reverse transcribed to cDNA using the high-capacity cDNA reverse transcription kit (Thermo Fisher Scientific, Waltham, MA, USA). The cDNA was subjected to quantitative reverse transcription polymerase chain reaction (qRT-PCR) using PowerUp™ SYBR™ Green Master Mix for qPCR (Cat# A25741, Applied Biosystems, Waltham, MA, USA). Gene expression levels were measured using human Bio-Rad PrimePCR™ SYBR^®^ Green assays for *GAPDH* (as a housekeeping gene) and CD206 (*MRC1*), *SLC16A3* (*MCT4*), *TNF-α*, *CXCL-10*, *IL-10*, *IL-1β*, *IL-6*, *IL-1Ra*, *HMOX-1*, PHD2 (*EGLN1*), *HIF-1α*, GLUT1 (*SLC2A1*), *PDK1*, *LDHB*, and *PDHA1*. The complete primer target names and biological functions are shown in Table 1, while Bio-Rad unique assay primer IDs are provided in Table S1. The primer sequences are confidential information that may be disclosed only by Bio-Rad Laboratories. The real-time PCR was performed on a 7500 Fast Real-Time PCR System (Applied Biosystems, Waltham, MA, USA). Relative mRNA expression levels were presented as (2^(−∆∆CT)^). Each PCR was performed in technical triplicate.

### 4.8. Immunofluorescent Staining of Intracellular CD206 in THP-1

THP-1 cells were cultured at a density of 5 × 10^5^ cells/2 mL in a Falcon™ 8-well Chambered Cell Culture Slide (Thermo Fisher Scientific, Waltham, MA, USA) and differentiated with PMA for 48 h. After adding fresh media, the differentiated macrophages were infected with MAP for 24 h, followed by treatment with MGO for another 24 h. Cells were washed with 1× cold phosphate-buffered saline (1× PBS) and then fixed on the slide with 10% formalin for 20 min. After fixation, the slides were washed twice with 1× PBS, then permeabilized with 0.2% Triton X-100 (Thermo Fisher Scientific, Waltham, MA, USA) for 10 min at room temperature. Cells were rinsed three times with 1× PBS for 5 min each, followed by cell blocking, which involved applying 100 µL of 10% goat serum blocking buffer (GS; Cat#50062Z, Invitrogen, Thermo Fisher Scientific) to each well and then incubating the cells in the dark for 1.30~2 h on a shaker. While blocking, a 1:100 primary antibody working solution was prepared by adding 98.9 µL of immunofluorescent goat serum blocking buffer, 0.1 µL of Tween-20, and 1 µL of CoraLite^®^Plus 488-conjugated CD206 monoclonal antibody (Proteintech, Rosemont, IL, USA; Cat.#CL488-60143). The blocking solution was aspirated, then 100 µL of antibody working solution was added to each well, and the slide was incubated overnight at 4 °C. Again, cells were rinsed four times in 1× PBS for 5 min each, and then the chamber was removed. The slide was allowed to dry for 20 min. After that, 4′,6-diamidino-2-phenylindole (DAPI; Vector Laboratories, Burlingame, CA, USA) was applied to stain the nuclei. Finally, the slides were examined under an AmScope fluorescent microscope (AmScope IN480 Series Inverted Epi-fluorescence Trinocular Compound Microscope, with a 6.0 Sony CCS sensor MF603C-CCD and 10× or 30× lens; AmScope, Irvine, CA, USA). The pictures were edited and merged using ImageJ software (Version 1.53k, National Institutes of Health, Bethesda, MD, USA), in which blue staining represents nuclei and green staining indicates CD206 signals. Images for each well were captured at 60×, magnified digitally, and analysis was performed as previously described by our lab [65]. The corrected total cell fluorescence (CTCF) of each image was calculated as follows: CTCF  =  integrated density − (area of selected cell  ×  mean fluorescence of background readings). The final fluorescence values were normalized to the untreated control group and expressed as the percentage fluorescence relative to the control.

### 4.9. ELISA Measurements of TNF-α, Nrf-2 and HIF-1α in MAP-Infected THP-1 Macrophages

Secreted TNF-α levels were quantified in cell culture supernatants collected from THP-1 macrophages following MAP infection and MGO treatment. Briefly, THP-1 macrophages were infected with MAP UCF4 for 24 h and then treated with the indicated concentrations of MGO for an additional 24 h. Culture supernatants were collected and centrifuged to remove cellular debris. TNF-α concentrations were measured using a Human TNF-alpha ELISA Kit (Cat. No. BMS223-4; Thermo Fisher Scientific/Bender MedSystems GmbH, Vienna, Austria) according to the manufacturer’s instructions. Absorbance was measured at 450 nm using a Multiskan FC plate reader (Thermo Fisher Scientific, Instruments Co., Ltd., Shanghai, China), and TNF-α concentrations were calculated from the standard curve. All samples were analyzed in triplicate, and data were expressed as mean ± SEM from three independent experiments.

Nrf-2 secretion and HIF-1α expression were analyzed 24 h after treating MAP-infected THP-1 with MGO. For Nrf-2, cell-free supernatants were collected, centrifuged, and quantified using the Invitrogen™ Human Nrf-2 ELISA Kit (EH348RBX5; Thermo Fisher Scientific, Waltham, MA, USA), following the kit protocol for culture supernatants. Absorbance was measured at 450 nm using a Multiskan FC plate reader.

For HIF-1α, adherent cells from parallel wells were washed twice with ice-cold PBS and lysed in RIPA buffer supplemented with protease and phosphatase inhibitors. Lysates were incubated on ice for 30 min and centrifuged at 12,000× *g* for 15 min at 4 °C. HIF-1α levels in the clarified lysates were determined using the Invitrogen™ Human HIF1A ELISA Kit (EHIF1A; Thermo Fisher Scientific, Waltham, MA, USA) according to the manufacturer’s instructions for cell lysates. Absorbance was recorded at 450 nm on the same plate reader. All conditions for both assays were run in triplicate.

### 4.10. Measurement of Lactate in MAP-Infected THP-1 Supernatants

To determine whether MGO alters lactate export in MAP-infected THP-1 macrophages, extracellular lactate levels were measured in culture supernatants. For lactate measurements, cells were maintained in medium containing dialyzed FBS (Thermo Fisher Scientific, Waltham, MA, USA) to minimize background lactate levels. THP-1 cells were differentiated with PMA and seeded in white 96-well plates at a density of 5 × 10^5^ cells/mL in 100 µL per well for 48 h. After differentiation, cells were assigned to the following groups: uninfected control, MAP-infected, and MAP-infected followed by 24 h of MGO treatment. Following the indicated treatment period, culture supernatants were collected, briefly centrifuged to remove cell debris, and assayed immediately. Extracellular lactate levels were measured using the Lactate-Glo™ Assay (J5021; Promega, Madison, WI, USA) according to the manufacturer’s instructions. Luminescence was recorded using a GloMax^®^ Navigator GM-2000 plate reader (Promega; Madison, WI, USA), and lactate levels were reported as relative luminescence units (RLU). Each experimental condition was performed in triplicate wells.

### 4.11. Pharmacological Interrogation of Nrf-2-Associated Antioxidant and MCT/Lactate-Linked Inflammatory Pathways in MAP-Infected THP-1 Macrophages

To evaluate whether Nrf-2-associated signaling contributes to the MGO-mediated antioxidant and anti-inflammatory response, PMA-differentiated THP-1 macrophages were infected with MAP UCF4 at 1 × 10^7^ CFU/mL for 24 h. Following infection, cells were washed with sterile PBS, and fresh culture medium was added. MAP-infected macrophages were pretreated with ML385 (Fisher Scientific, supplied by Selleck Chemicals LLC (Houston, TX, USA; supplier Cat. # S87905MG; Fisher Cat. # 50-194-8057; CAS# 846557-71-9)), an Nrf-2 pharmacological inhibitor, at a final concentration of 5 µM for 3 h, then treated with 50 µg/mL MGO for 24 h. N-acetyl-L-cysteine (NAC; Sigma-Aldrich, St. Louis, MO, USA; Cat. # A9165; CAS # 616-91-1) was used as an antioxidant positive control at a final concentration of 10 mM. Experimental groups included untreated control, MAP, MAP + ML385, MAP + ML385 + MGO, MAP + MGO, and MAP + NAC.

To examine the contribution of monocarboxylate transporter/lactate-linked signaling to the MGO-mediated response, MAP-infected THP-1 macrophages were treated with 0.25 mM α-cyano-4-hydroxycinnamic acid (α-CHC; MilliporeSigma, Burlington, MA, USA; Cat. #C2020; CAS# 28166-41-8), a broad-spectrum monocarboxylate transporter inhibitor, prior to MGO treatment. Experimental groups included untreated control, MAP, MAP + MGO, MAP + CHC, and MAP + CHC + MGO. Appropriate vehicle controls were included for ML385 and α-CHC. Following treatment, cells were collected for RNA extraction, cDNA synthesis, and qRT-PCR analysis of IL-6 or TNF-α expression. Gene expression was normalized to GAPDH and calculated using the 2^−ΔΔCt^ method.

### 4.12. MGO Pretreatment and MCT4 Inhibition in MAP-Infected THP-1

To evaluate whether MGO pretreatment exerts a protective anti-inflammatory effect against MAP-induced macrophage activation, PMA-differentiated THP-1 macrophages were pretreated with 50 µg/mL MGO for 3 h before MAP infection. In parallel, to assess whether inhibition of monocarboxylate transporter activity attenuates MAP-induced inflammatory signaling, cells were pretreated with 0.25 mM α-cyano-4-hydroxycinnamic acid (α-CHC), a broad monocarboxylate transporter inhibitor [66], for 3 h before infection. α-CHC stock solution was prepared in methanol and stored as directed by the manufacturer. Appropriate methanol vehicle controls were included for α-CHC-treated groups. After pretreatment, THP-1 macrophages were infected with MAP (UCF4) at 1 × 10^7^ CFU/mL for 24 h. During the post-infection period, cells were collected for RNA extraction, followed by cDNA synthesis and qRT-PCR analysis of *TNF-α* expression. Gene expression was normalized to GAPDH, and relative expression was calculated using the 2^−ΔΔCt^ method.

### 4.13. Detection and Quantitation of Methyl-Glyoxal-Hydro-Imidazolone (MG-H1)

MGO acts as a hormetic agent, inducing adverse effects at extremely elevated intracellular concentrations [67]. To confirm the safety dose of 50 μg/mL MGO, MG-H1 levels were measured using the OxiSelect™ Methylglyoxal Competitive ELISA Kit (Cat. # STA-811, Cell Biolabs, San Diego, CA, USA) according to the manufacturer’s instructions. Briefly, THP-1 macrophages were seeded at 5 × 10^5^ cells/well in RPMI medium in a 12-well tissue culture plate and differentiated. Then, THP-1 macrophages were infected with MAP UCF4 (1 × 10^7^ CFU/mL) for 24 h. Subsequently, cells were treated with 50 μg/mL MGO for 24 h. Statistical analyses were performed using GraphPad Prism. All experiments were conducted using biological triplicates (*n* = 3 per group).

### 4.14. Statistical Analysis

All experiments were conducted using biological triplicates (*n* = 3 per group). Statistical analyses were performed using GraphPad Prism version 10.4.4 (GraphPad Software, Boston, MA, USA). Data distribution was assessed using the Shapiro–Wilk normality test. For comparisons involving more than two groups, a Kruskal–Wallis nonparametric one-way ANOVA was performed, followed by Dunn’s post hoc multiple-comparisons test to compare MAP and MAP + 50 μg/mL MGO-treated groups with the control group. When data satisfied normality assumptions, one-way or two-way analysis of variance (ANOVA) was performed as appropriate, followed by Dunnett’s or Bonferroni post hoc multiple-comparison tests. Results are expressed as mean ± standard deviation (SD). Statistical significance was defined as * *p* < 0.05, ** *p* < 0.01, *** *p* < 0.001, and **** *p* < 0.0001 was considered significant.

## 5. Conclusions

**Figure 11:**
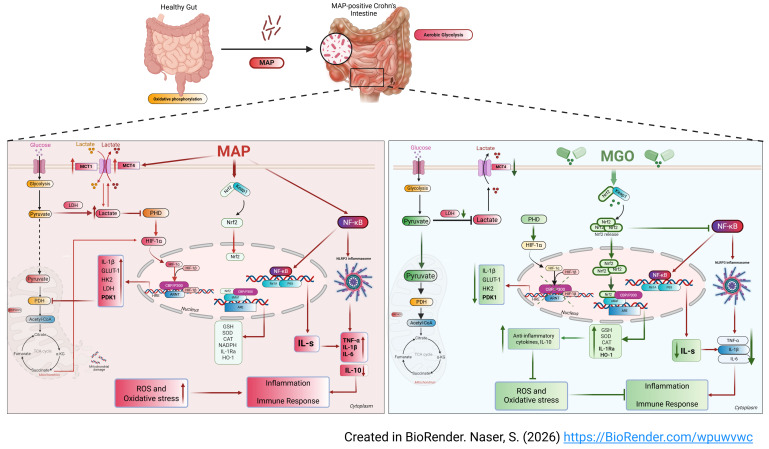
Hormetic dosage of methylglyoxal as a strong brake on MAP-driven inflammatory polarization by engaging the Nrf-2/HO-1 defense program and shifting immune-cell metabolism away from a pro-inflammatory state.

Collectively, this study provides in vitro proof-of-concept evidence that a hormetic dose of MGO may represent a naturally derived adjunctive candidate to reduce MAP-induced macrophage inflammation and modulate selected antioxidant and glycolysis- and lactate-associated inflammatory markers in MAP-associated CD models (Figure 11). Our findings highlight the hormetic dose of methylglyoxal (MGO) as a strong brake on MAP-driven inflammatory polarization by engaging the Nrf-2 defense program, reducing inflammatory cytokine expression, and shifting glycolysis- and lactate-associated markers away from the pro-inflammatory pattern. Together, these findings support further preclinical evaluation of MGO as an immunometabolic candidate for MAP-associated CD, with future studies needed to validate its efficacy and safety in animal models and translational settings. We believe that MGO is a promising therapeutic target for MAP-associated CD.

## 6. Limitations and Future Directions

One limitation of this study is that classical macrophage polarization controls were not included. Future studies will include LPS/IFN-γ-treated cells as M1-positive controls and IL-4-treated cells as M2-positive controls, together with expanded marker analysis, to more rigorously define the macrophage polarization state induced by MAP and MGO. Although *CXCL10* and CD206 (*MRC1*) are informative M1/M2 markers, macrophage polarization is more accurately assessed using a broader marker panel. Future studies will include additional M1-associated markers, including iNOS, CD80, CD86, IL-12, and HLA-DR, as well as M2-associated markers, including ARG1, CD163, IL-4Rα, CCL18, and YM1. This expanded analysis will provide a more complete characterization of macrophage phenotype and will help define how MAP infection and MGO treatment influence macrophage inflammatory and anti-inflammatory responses. Future studies should validate these findings in primary human macrophages and appropriate animal models, examine NRF2 nuclear translocation and downstream transcriptional activity, and investigate additional upstream pathways regulating the anti-inflammatory and metabolic effects of MGO.

Although MGO reduced several HIF-1α-associated downstream targets, including *GLUT1*, *IL-1β*, and *PDK1*, the present study did not directly determine whether this effect was mediated through FIH activity, altered HIF-1α hydroxylation, post-translational modification, changes in HIF-1α protein half-life, or reduced HIF-1α/p300 coactivator recruitment. Future studies are required to examine these mechanisms by measuring FIH activity, assessing hydroxylated *HIF-1α*, evaluating HIF-1α protein stability/half-life, and performing co-immunoprecipitation of HIF-1α with p300/CBP. These experiments will clarify whether MGO suppresses *HIF-1α* transcriptional activity independently of changes in total *HIF-1α* expression in MAP-infected macrophages. In addition, future studies are needed to determine whether MGO affects OXPHOS or mitochondrial respiratory function. Compared with well-known anti-inflammatory metabolites and byproducts, MGO needs to be overlooked. Although MGO reduced several glycolysis-associated markers, including *GLUT1*, *PDK1*, *LDHB*, MCT4 (SLC16A3), and lactate export, the present study did not directly measure mitochondrial respiration. Future studies are needed to incorporate Seahorse XF analysis to measure the oxygen consumption rate (OCR) and extracellular acidification rate (ECAR), and to more precisely define how MGO affects glycolytic and mitochondrial activity in MAP-infected macrophages. Mitochondrial function should also be evaluated by measuring ATP production, the ATP/ADP ratio, and mitochondrial membrane potential using JC-1 and TMRE assays. In light of the observed Nrf-2 activation, future studies need to examine whether MGO affects the pentose phosphate pathway (PPP) by assessing G6PD expression or activity, measuring the NADPH/NADP^+^ ratio, quantifying ribose-5-phosphate, and performing ^13^C-glucose tracing to track carbon flow. These studies will help determine whether MGO redirects macrophage metabolism from inflammatory glycolytic activity toward redox maintenance and, consequently, mitochondrial metabolic recovery.

Another limitation of the present study is the use of the PMA-differentiated THP-1 macrophage model, which provides a reproducible in vitro system but does not fully recapitulate the complexity of primary human macrophages or the intestinal immune microenvironment. To address this limitation, future studies will validate these findings in primary human macrophages and relevant in vivo models that more closely reflect MAP-associated intestinal inflammation. Building on the current macrophage findings, we are now extending this work to intestinal epithelial cell models to further address epithelial–immune interactions and translational relevance.

## Figures and Tables

**Table 1:** Primers for quantitative real-time PCR targets and biological function used in this study.

Abbreviation	Primer Name	Biological Marker/Function	Source
CD206 (*MRC1*)	Mannose receptor C-type 1	M2 macrophage marker; anti-inflammatory and tissue-repair phenotype	Bio-Rad, Hercules, CA, USA
*CXCL-10*	C-X-C motif chemokine ligand 10	Pro-inflammatory chemokine; M1 Marker.	Bio-Rad, Hercules, CA, USA
GAPDH	Glyceraldehyde-3-phosphate dehydrogenase	Housekeeping gene; glycolytic enzyme used for normalization	Bio-Rad, Hercules, CA, USA
GLUT1 (*SLC2A1*)	Solute carrier family 2 member 1	Glucose transporter; marker of glycolytic activation	Bio-Rad, Hercules, CA, USA
*HIF-1α*	Hypoxia-inducible factor-1 alpha	Transcription factor regulating glycolysis and inflammatory metabolism	Bio-Rad, Hercules, CA, USA
HMOX-1 (*HO-1*)	Heme oxygenase-1	Nrf-2 target gene; oxidative stress response and cytoprotection	Bio-Rad, Hercules, CA, USA
*IL-10*	Interleukin-10	Anti-inflammatory cytokine; immune regulation and tolerance	Bio-Rad, Hercules, CA, USA
*IL-1Ra* (*IL-1RN*)	Interleukin-1 receptor antagonist	Anti-inflammatory cytokine; antagonist of IL-1 signaling	Bio-Rad, Hercules, CA, USA
*IL-1β*	Interleukin-1 beta	Pro-inflammatory cytokine; inflammasome-dependent response	Bio-Rad, Hercules, CA, USA
*IL-6*	Interleukin-6	Pro-inflammatory cytokine; metabolic inflammation	Bio-Rad, Hercules, CA, USA
*LDHB*	Lactate dehydrogenase B	Lactate–pyruvate recycling	Bio-Rad, Hercules, CA, USA
*PDHA1*	Pyruvate dehydrogenase E1 alpha subunit	Links glycolysis to TCA; mitochondrial oxidative metabolism	Bio-Rad, Hercules, CA, USA
*PDK1*	Pyruvate dehydrogenase kinase 1	Inhibits PDH; shifts metabolism toward glycolysis	Bio-Rad, Hercules, CA, USA
PHD2 (*EGLN1*)	Prolyl hydroxylase domain protein 2	Oxygen sensor; promotes HIF-1α ubiquitination and degradation	Bio-Rad, Hercules, CA, USA
SLC16A3 (MCT4)	Solute carrier family 16 member 3	Lactate exporter; marker of glycolytic and inflammatory metabolism	Bio-Rad, Hercules, CA, USA
TNF-α	Tumor necrosis factor alpha	Pro-inflammatory cytokine; NF-κB-mediated inflammation	Bio-Rad, Hercules, CA, USA

## Data Availability

The original contributions presented in this study are included in the article. Further inquiries can be directed to the corresponding author.

## Supplementary Materials

The following supporting information can be downloaded at: https://www.mdpi.com/article/10.3390/ijms27156940/s1.

## Abbreviations

The following abbreviations are used in this manuscript:

ATCC	American Type Culture Collection
CBP/p300	CREB-binding protein/p300 coactivator
CD	Crohn’s disease
CD206	Cluster of differentiation 206; macrophage mannose receptor
cDNA	complementary DNA
CFU	Colony-forming unit
α-CHC	α-cyano-4-hydroxycinnamic acid
CXCL-10	C-X-C motif chemokine ligand-10
DAPI	4′,6-diamidino-2-phenylindole
ELISA	Enzyme-linked immunosorbent assay
FBS	Fetal bovine serum
FIH	Factor inhibiting HIF
GAPDH	Glial fibrillary acidic protein
GLUT1	Glucose transporter 1
GSH	Reduced glutathione
GU	Growth units
HIF-1α	Hypoxia-inducible factor 1 alpha
HK2	Hexokinase isoform
HO-1	Heme oxygenase-1
HRE	Hypoxic response elements
IBD	Inflammatory bowel disease
IL-1β	Interleukin-1β
IL-1RN	Interleukin-1 receptor antagonist
IL-6	Interleukin 6
IL-10	Interleukin-10
Keap1	Kelch-like ECH-associated protein 1
LDH	Lactate dehydrogenase
MAP	*Mycobacterium avium paratuberculosis*
MCT4	Monocarboxylate transporters 4
MGIT	Mycobacteria growth indicator tube
MGO	Methylglyoxal
NAC	N-acetyl-L-cysteine
NADPH	Nicotinamide adenine dinucleotide phosphate (reduced form)
NF-κB	Nuclear factor-kappa B
Nrf-2	Nuclear factor erythroid 2-related factor 2
OXPHOS	Oxidative phosphorylation
PBS	Phosphate-buffered saline
PDH	Pyruvate dehydrogenase
PDK1	Pyruvate dehydrogenase kinase 1
PHD2	Prolyl hydroxylase domain-containing protein 2
PMA	Phorbol 12-myristate 13-acetate
RCF	Relative centrifugal force
RLU	Relative luminescence units
ROS	Reactive oxygen species
RT-qPCR	Reverse transcription-quantitative polymerase chain reaction
SEM	Stranded error of the mean
TCA	Tricarboxylic acid
TLR-2/4	Toll-like receptor-2/4
TNF-α	Tumor necrosis factor alpha
UC	Ulcerative colitis
VHL	von Hippel–Lindau
