# Supplementary material for: Methylglyoxal Attenuates Mycobacterium avium subspecies paratuberculosis (MAP)-Induced Pro-Inflammatory Macrophage Programming Associated with NRF-2 Antioxidant Responses and Reduced MCT4/Lactate-Linked Inflammatory Markers
Source: Int J Mol Sci. 2026 Aug 2;27(15):6940. doi: 10.3390/ijms27156940 (PMC13467399; doi:10.3390/ijms27156940)
Supplement: Supplementary file 1 [file ijms-27-06940-s001.zip › ijms-4431626-supplementary.pdf]

Supplementary Materials

Article title: Methylglyoxal Attenuates *Mycobacterium avium subspecies paratuberculosis* (MAP)-Induced Pro-Inflammatory Macrophage Programming Associated with NRF-2 Antioxidant Responses and Reduced MCT4/Lactate-Linked Inflammatory Markers

Authors: Heba R. AlRefaey [1], and Saleh A. Naser [1,*]

Table S1. Bio-Rad PrimePCR™ SYBR® Green assays used for quantitative real-time PCR, including gene targets and unique assay IDs.

Abbreviation	Primer name	Source	Bio-Rad PrimePCR unique assay ID
CD206 (MRC1)	Mannose receptor C-type 1	Bio-Rad, Hercules, CA, USA	qHsaCID0037074
CXCL-10	C-X-C motif chemokine ligand 10	Bio-Rad, Hercules, CA, USA	qHsaCED0046619
GAPDH	Glyceraldehyde-3-phosphate dehydrogenase	Bio-Rad, Hercules, CA, USA	qHsaCED0038674
GLUT1 (SLC2A1)	Solute carrier family 2 member 1	Bio-Rad, Hercules, CA, USA	qHsaCED0043733
HIF-1α	Hypoxia-inducible factor-1 alpha	Bio-Rad, Hercules, CA, USA	qHsaCID0014755
HMOX-1 (HO-1)	Heme oxygenase-1	Bio-Rad, Hercules, CA, USA	qHsaCID0022141
IL-10	Interleukin-10	Bio-Rad, Hercules, CA, USA	qHsaCED0003369
IL-1Ra (IL-1RN)	Interleukin-1 receptor antagonist	Bio-Rad, Hercules, CA, USA	qHsaCED0044706
IL-1β	Interleukin-1 beta	Bio-Rad, Hercules, CA, USA	qHsaCID0022272
IL-6	Interleukin-6	Bio-Rad, Hercules, CA, USA	qHsaCID0020314
LDHB	Lactate dehydrogenase B	Bio-Rad, Hercules, CA, USA	qHsaCED0043397

Abbreviation	Primer name	Source	Bio-Rad PrimePCR unique assay ID
PDHA1	Pyruvate dehydrogenase E1 alpha subunit	Bio-Rad, Hercules, CA, USA	qHsaCED0001525
PDK1	Pyruvate dehydrogenase kinase 1	Bio-Rad, Hercules, CA, USA	qHsaCID0009131
PHD2 (EGLN1)	Prolyl hydroxylase domain protein 2	Bio-Rad, Hercules, CA, USA	qHsaCED0057071
SLC16A3 (MCT4)	Solute carrier family 16 member 3	Bio-Rad, Hercules, CA, USA	qHsaCID0014322
TNF-α	Tumor necrosis factor alpha	Bio-Rad, Hercules, CA, USA	qHsaCED0037461
